# Nanoconfinement Engineering of Covalent Organic Frameworks in Polyamide Membranes for High‐Perselectivity Li^+^/Mg^2+^ Separation

**DOI:** 10.1002/advs.202500255

**Published:** 2025-04-02

**Authors:** Shuzhen Zhao, Liheng Dai, Zhaohuan Mai, Bowen Li, Pengfei Zhang, Mengxiao Zhang, Atsushi Matsuoka, Kecheng Guan, Ryosuke Takagi, Hideto Matsuyama

**Affiliations:** ^1^ Research Center for Membrane and Film Technology Kobe University 1‐1 Rokkodaicho, Nada Kobe 657–8501 Japan; ^2^ Department of Chemical Science and Engineering Kobe University 1‐1 Rokkodaicho, Nada Kobe 657–8501 Japan; ^3^ MOE Key Laboratory of Macromolecular Synthesis and Functionalization Department of Polymer Science and Engineering Zhejiang University Hangzhou 310027 China

**Keywords:** covalent organic framework, interfacial polymerization, Li^+^/Mg^2+^ separation, nanoconfinement regulation

## Abstract

Efficiently separating Li^+^ over Mg^2+^ from brines remains a significant challenge due to their minimal size difference of only several angstroms, posing a substantial difficulty for traditional nanofiltration (NF) membranes. Therefore, a nanoconfinement regulation strategy utilizing a porous covalent organic framework (COF) layer is proposed to precisely control the interfacial polymerization (IP) process, thereby obtaining a thin, uniform polyamide (PA) membrane for high‐efficiency Li^+^/Mg^2+^ separation. The nanoconfinement microenvironment fundamentally modulates both monomer spatial distribution and reaction kinetics through porous confined space and abundant interaction sites provided by the COF layer. Consequently, the resulting TpPa‐S/PA membrane exhibits a narrower pore size distribution, achieving an ion sieving precision of 0.46 Å. Due to the strict size sieving effect, the Li^+^/Mg^2+^ separation factor exceeded 120, which is one to two orders of magnitude higher than all the currently reported NF membranes. This study addressed the typical limitations inherent in conventional NF membranes, establishing a promising foundation for advancing lithium extraction technologies through nanoconfinement strategy‐regulated IP processes.

## Graphical abstract

A nanoconfinement strategy using porous COF layers is developed to regulate interfacial polymerization and prepare thin, uniform PA membranes. The COF layer provides confined spaces and abundant interaction sites, effectively controlling monomer distribution and reaction kinetics. This approach yields a PA membrane with a narrow pore size distribution and a Li^+^/Mg^2+^ separation factor exceeding 120, significantly outperforming conventional nanofiltration membranes.

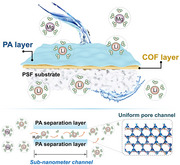

## 1 Introduction

The rapid global sustainable development has driven an unprecedented demand for lithium (Li), necessitating the advancement of efficient extraction technologies to ensure long‐term supply sustainability.^[^ ^1^ ^]^ Salt lake brines, which account for ≈70% of the world's Li reserves, are a promising resource.^[^ ^2^ ^]^ However, the extraction of Li⁺ from these brines exists significant challenges due to their complex ionic composition, particularly the high concentrations of magnesium ions (Mg^2+^), which typically exceed those of Li^+^ by more than twentyfold.^[^ ^3^ ^]^ Furthermore, the close similarity in hydrated ionic radii between Li^+^ (3.8 Å) and Mg^2+^ (4.3 Å) further complicates separation, making conventional methods inadequate for effective lithium recovery.^[^ ^4^, ^5^ ^]^

Among various emerging separation technologies, nanofiltration (NF) membrane technology has obtained significant attention due to its energy‐saving advantage and superior selectivity in ion separation.^[^ ^6^, ^7^ ^]^ Current commercial NF membranes are polyamide (PA) typically fabricated via interfacial polymerization (IP) process which can create thin‐film composite membranes with nanoscale pore structures.^[^ ^8^, ^9^ ^]^ However, the rapid interfacial reaction kinetics tends to result in non‐uniform pore size distributions and structural defects,^[^ ^10^ ^]^ which significantly reduce the sieving precision especially for Li^+^/Mg^2+^ system only with several angstrom differences. Addressing the above challenges, surface charge engineering such as fabricating strong positive charged NF membrane based on the Donnan effect could effectivity repulse Mg^2+^ but it also increases rejection of Li.^+[^ ^11^, ^12^ ^]^ Optimizing the pore size of NF membrane is an effective method to sieve Li^+^ over Mg^2+^ according to the size‐exclusion mechanism, which may also break the limitation of charge repulsion. Precisely designing the membrane pore size to match the size of Li^+^ and Mg^2+^ is full of challenges, where controlling IP reaction process is crucial to achieve the formation of uniform pores and narrow pore size distribution.^[^ ^13^ ^]^ Unfortunately, research in this area is still immature and there are few relevant reports. Adding water‐soluble^[^ ^14^ ^]^ or oil‐soluble surfactants^[^ ^15^ ^]^ could conduce to the formation of ordered monolayers at the interface to further manipulate the IP process, but it required more accurate chemical dosage and finely tuned experiment skills.

Recently, nanoconfinement technology offered a promising solution by providing a well‐nanoscale confined or interaction space, expected to restrict monomer diffusion and reaction, thereby enabling unprecedented control over catalysis reaction, interfacial force, and polymerization uniformity.^[^ ^16^, ^17^, ^18^ ^]^ Nanoconfinement is fundamentally a dynamic regulation mechanism driven by the synergistic effect between spatially restricted environments and intermolecular interactions.^[^ ^19^ ^]^ When molecules are confined within host materials at sub‐nanometer to nanometer scales, the combined effects of physical confinement and the chemical microenvironment collectively govern the transport, distribution, and reactivity of guest molecules.^[^ ^20^, ^21^ ^]^ These confinement‐induced effects lead to properties that differ significantly from those observed in pristine systems, thereby providing a distinctive platform for the precise manipulation of molecular behavior.^[^ ^22^ ^]^ In this context, covalent organic frameworks (COFs) have emerged as particularly promising candidates for implementing nanoconfinement strategies. Their unique combination of tunable pore sizes, functional versatility, and robust covalent‐bonded structures^[^ ^23^, ^24^, ^25^ ^]^ provides an ideal platform for optimizing the IP process. The highly ordered microporous structure of COFs creates naturally nanoconfined environments that regulate monomer diffusion and reaction dynamics with exceptional precision.^[^ ^26^ ^]^ Moreover, COFs can be synthesized directly via IP method, which not only simplifies the preparation procedure but also provides a versatile platform for subsequent regulation of the membrane properties.^[^ ^27^, ^28^ ^]^ By creating a nanoconfined environment within the COF layer, the diffusion and reaction kinetics of monomers are precisely controlled, resulting in enhanced uniformity of polymerization, reduced defects, and consistent pore structures, resulting in NF membranes with superior separation performance.

**Figure 1:**
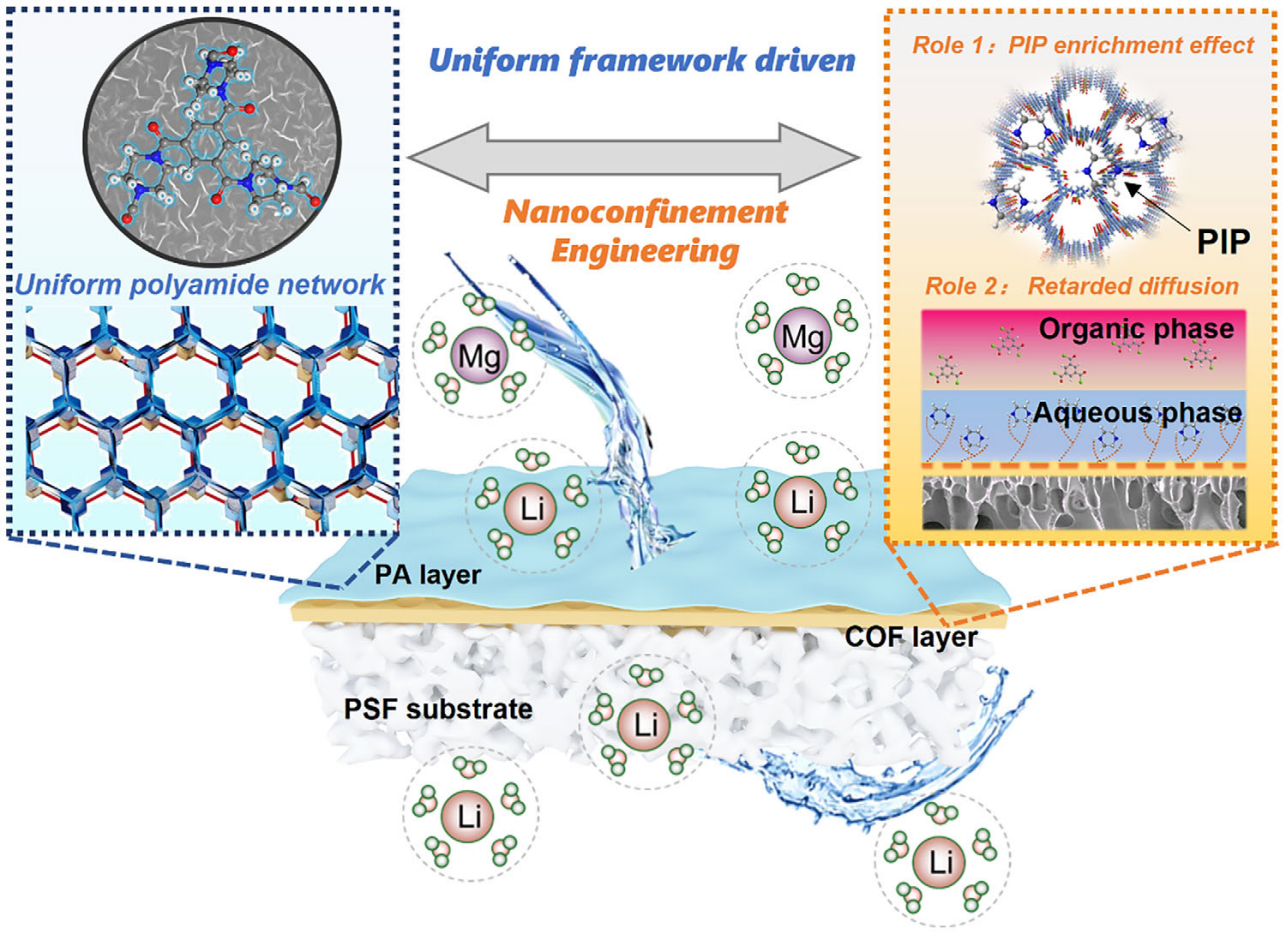
Schematic diagram of the PA layer with uniform pores and corresponding interfacial polymerization regulation process via the nanoconfinement mechanism from the COF layer.

Herein, we employed a novel nanoconfinement regulation mechanism to control IP process to further reconstruct the physicochemical properties of NF membranes, thereby improving the Li⁺/Mg^2^⁺ separation selectivity. Specifically, a porous COF nanofilm with abundant sulfonic acid groups was in situ synthesized on polysulfone (PSF) substrates, resulting in a highly controlled nanoconfined microenvironment, as illustrated in **Figure** **1**. The nanoconfined microenvironment significantly enriched PIP monomers at the interface, ensured their uniform spatial distribution, and precisely regulated their diffusion into the organic phase, achieving fine control of the interfacial reaction. A thinner, denser PA active layer with a narrower pore size distribution was designed and obtained under the nanoconfinement regulation mechanism. Such a refined membrane structure greatly improved the size‐sieving capability of the membrane, thereby facilitating more efficient differentiation between Mg^2^⁺ and Li⁺ with similar hydrated radii. As a result, the PA membranes exhibited a significant improvement in Li⁺/Mg^2^⁺ selectivity, while simultaneously exhibiting a remarkable increase in permeability compared to conventional NF membranes. The robustness and long‐term operational stability of these membranes highlight their potential for industrial‐scale Li⁺ extraction from salt lake brines.

## 2 Results and Discussion

**Figure 2:**
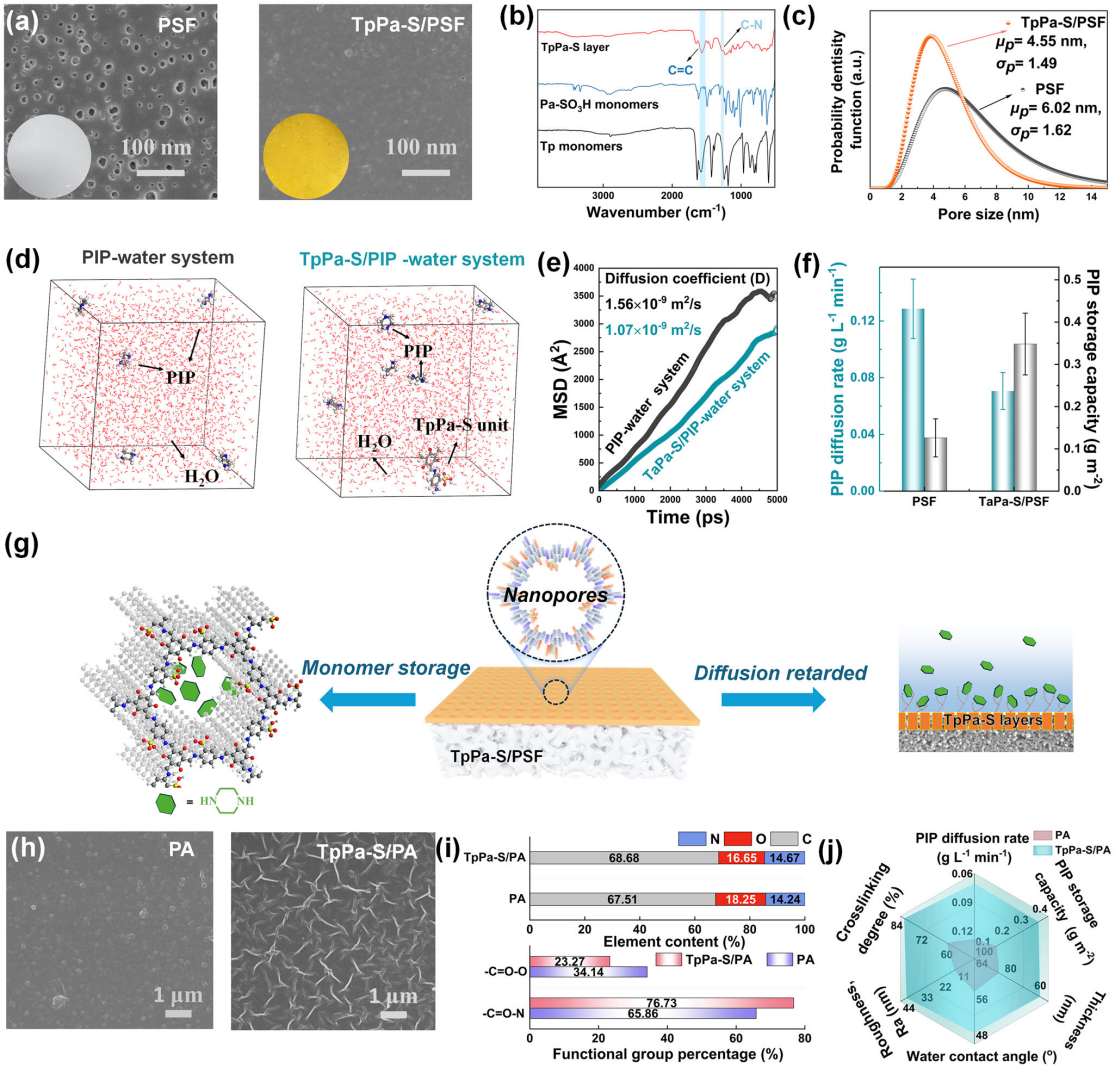
Nanoconfinement regulation mechanism of the COF layer a) SEM surface and digital photos, b) ATR‐IR spectra, c) pore size distribution of substrates with and without TpPa‐S nanofilm; d) molecular dynamics simulations illustrations of PIP behavior in the PIP‐water system and PIP/TpPa‐S‐water system; e) mean square displacement and diffusion coefficients of PIP molecules in both the PIP‐water and PIP/TpPa‐S‐water systems; f) the diffusion rates of PIP into n‐hexane and PIP adsorption capacity on PSF substrates with and without TpPa‐S nanofilm; g) schematic diagram of the key roles of the TpPa‐S layer in the regulation of PIP monomers; h) SEM surface images, i) elemental and functional group composition, j) comparison of various factors and properties of PA membranes with and without TpPa‐S nanofilm control.

First, a continuous and defect‐free TpPa‐S COF nanofilm was directly constructed on the macroporous PSF substrate via an in situ interfacial Schiff base process between 2,4,6‐Triformylphloroglucinol (Tp) and 2,5‐Diaminobenzenesulfonic acid (Pa‐SO_3_H) monomers (Figure S1, Supporting Information; **Figure** **2**a). The successful growth of the TpPa‐S COF layer was confirmed by Attenuated Total Reflectance‐Fourier Transform Infrared Spectroscopy (ATR‐FTIR) analysis, as evidenced by the appearance of characteristic peaks at 1278 and 1573 cm^−1^, corresponding to the C─N and C = C bonds formed during the Schiff base reaction (Figure 2b).^[^ ^26^, ^29^ ^]^ In addition, the hydrophilicity of the PSF substrate was significantly enhanced due to the presence of the TpPa‐S layer, as indicated by a reduction in the water contact angle from 82.1° to 60.3° (Figure S2, Supporting Information). After growing the TpPa‐S COF layer, the molecular weight cut off (MWCO) of the membranes decreased from 137.5 kDa for the pristine PSF substrate to 90.5 kDa for the TpPa‐S COF modified membrane (Figure S3, Supporting Information), while the corresponding average pore size of membrane decreased from 6.02 to 4.55 nm (Figure 2c). Notably, the designed TpPa‐S COF layer did not significantly sacrifice the pure water flux of PSF substrate (Figure S2, Supporting Information). This could be attributed to the well‐defined and uniform pore structure of the TpPa‐S,^[^ ^30^ ^]^ combined with the increased surface hydrophilicity, both of which contribute to maintaining efficient water permeability. Thus, the structural and chemical modifications introduced by the TpPa‐S COF layers not only create a novel membrane surface microenvironment with unique physicochemical properties but also preserve its water transport capacities, supporting the formation of a high‐performance separation layer.^[^ ^31^ ^]^ Although achieving efficient Li^+^/Mg^2+^ separation within such a pore environment remains a challenge for most pressure‐driven COF membranes, it is worth noting that TpPa‐S COF layer with nanopores created a unique physicochemical environment which could offer a valuable platform for further nanoconfined surface modification and reconstruction of the membrane. To enhance the membrane separation ability and precision, a dense PA layer was subsequently engineered. Importantly, the nanoporous structure of TpPa‐S COF layer is expected to manipulate the growth kinetics of the PA layer from the perspective of interfacial chemistry, enabling precise control over the structure and separation performance of the PA layers.

The key to achieving an ordered and uniform PA structure for ultra‐permeability and precise separation lies in finely controlling the IP process, especially for diffusion behavior and reaction dynamics.^[^ ^32^ ^]^ To understand the nanoconfinement roles of TpPa‐S toward PIP as monomers for PA layer formation, we investigated the diffusion behavior of PIP under the absence and existence of TpPa‐S system via molecular dynamics (MD) simulations (Figure 2d). Specifically, in the PIP/TpPa‐S‐water system, the diffusion coefficient was significantly reduced to 1.05 × 10^−9^ m^2^ s^−1^, lower than in the PIP‐water system (1.57 × 10^−9^ m^2^ s^−1^), indicating that the TpPa‐S layer imposes a notable restriction on PIP mobility (Figure 2e).^[^ ^33^ ^]^ Furthermore, we used Ultraviolet–visible (UV–vis) spectroscopy to determine the PIP diffusion rate from the water phase to the organic phase,^[^ ^34^ ^]^ which was slowed to 0.0706 g L^−1^ min^−1^ after introducing TpPa‐S layer. The results are also consistent with the above simulation results. Meanwhile, we observed there was a markedly higher PIP adsorption capacity (0.348 g m^−2^) for TpPa‐S/PSF membrane compared to the pristine PSF substrate (0.126 g m^−2^) (Figure 2f). It can be seen that TpPa‐S layer played an important role in controlling the diffusion process of PIP and storage behavior in the membrane surface. As shown in Figure S4 (Supporting Information), TpPa‐S layers have abundant nanopores and functional side‐chain groups, and meanwhile, the surface zeta potential result showed that the TpPa‐S layer was negatively charged. Additional nanopores could provide more interaction sites and space for PIP molecules to manipulate their diffusion and storage behavior, and meanwhile, functional groups further strengthen the nanoconfinement process. To further validate the crucial role of the nanoporous structure in regulating the IP process, a TpPa COF layer based on Tp and p‐phenylenediamine (Pa) monomers is designed and fabricated following the same method (Figure S5, Supporting Information). Similarly, the PIP diffusion behavior and storage capacity are determined and compared based on the TpPa/PSF system. As shown in Figure S6 (Supporting Information), the introduction of the TpPa layer effectively reduced the PIP diffusion rate while simultaneously enhancing its storage capacity compared to the PSF substrate. Notably, the nanoconfinement effect of TpPa is found to be weaker than that of the TpPa‐S system, suggesting that while the nanoporous structure of the COF layer plays a fundamental role in nanoconfinement regulation during the IP process, while a well‐defined microchemical environment further strengthens this effect. This is an important reason to choose the TpPa‐S as an activated interlayer. Therefore, the nanoconfinement regulation toward PIP monomers by the TpPa‐S layer during IP process is mainly reflected in two aspects (Figure 2g): i) enriching the PIP monomers on the substrate surface and ii) ensuring their uniform distribution and limiting the disordered and rapid diffusion of PIP monomers through interactions between the TpPa‐S layer and the PIP, thereby promoting a controlled polymerization process.

Based on the nanoconfinement regulation process of TpPa‐S layer, a PA active layer without visible defect was further formed on the surface of TpPa‐S/PSF membrane, whose chemical structure was confirmed by the ATR‐FTIR, X‐ray photoelectron spectroscopy (XPS) spectra (Figure S7, Supporting Information). Energy Dispersive X‐ray Spectroscopy (EDS) mapping results also confirm a continuous PA layer completely covers the COF layer surface. (Figure S8 and Table S1, Supporting Information). Compared with pristine PA membrane, under the manipulation of TpPa‐S layer, the membrane surface morphologies achieved the change from a typical nodular morphology to a regular nano‐ridged, wrinkled morphology (Figure 2h). This morphology change was also observed by Atomic Force Microscopy (AFM), where the membrane roughness increased from 12.3 to 65.9 nm (Figure S9 and Table S2, Supporting Information). The transformation in surface morphology, which was directly related to the modulatory influence of the TpPa‐S layers on the IP process, arises from the interactions between the TpPa‐S layer and PIP monomers (hydrogen bonding, electrostatic attraction, and physical confinement). The nanoconfinement effect amplifies the diffusion rate disparity between the two‐phase monomers, inducing instability at the oil–water interface and ultimately leading to the formation of a uniform nano wrinkle morphology.^[^ ^35^, ^36^ ^]^ According to Freger's model,^[^ ^37^ ^]^ a slower diffusion process of PIP from the aqueous phase to the organic phase, coupled with its higher concentration near the interface, thereby synchronizing the monomer availability and reaction kinetics at the interface, leads to a thinner and more controlled separation layer. Correspondingly, as shown in Figure S10 (Supporting Information), PA layer thickness decreased from 80.7 to 54.2 nm, which will lead to decreasing mass transfer resistance. It can be seen that this nanoconfinement regulation mechanism of TpPa‐S layer plays a crucial role in modulating the IP dynamics, expected to facilitate the formation of a more controlled and uniform PA layer.

In addition, the chemical composition of the PA layer under the manipulation of the TpPa‐S layer was finely reconstructed (Figure 2i), where there was a significant increase in the proportion of N/O and ─N─C = O bonds and corresponding the proportion of ─O─C = O bonds decreased. Notably, the ─N─C = O bond is integral to the polyamide structure, whereas the presence of ─O─C = O bonds generally indicated the hydrolysis of unreacted acyl chloride groups. Therefore, a higher ratio of ─N─C = O bonds suggested an increased cross‐linking degree within the polyamide network,^[^ ^38^ ^]^ which was expected to achieve the construction of PA layer with uniform channels. Meanwhile, the reduction of the ─COOH groups resulted in a weakened negative charge on the membrane surface (Figure S11, Supporting Information). It can be seen that IP process regulated by nanoconfinement engineering of the TpPa‐S layer effectively promoted the formation of a thinner separation layer with higher crosslinking density (Figure 2j; Figure S12 and Table S3, Supporting Information), exhibiting improved surface roughness and enhanced hydrophilicity (Figure S13, Supporting Information), which will reconstruct the PA layer to achieve the improvement of separation accuracy. Similarly, under nanoconfinement regulation, the resulting TpPa/PA membrane also exhibits characteristic wrinkled surface morphologies and a thinner membrane thickness, along with an increased degree of crosslinking in the PA layer (Figures S14 and S15, Supporting Information), which further confirm the key role of nanoconfinement effect on formation of PA layer.

**Figure 3:**
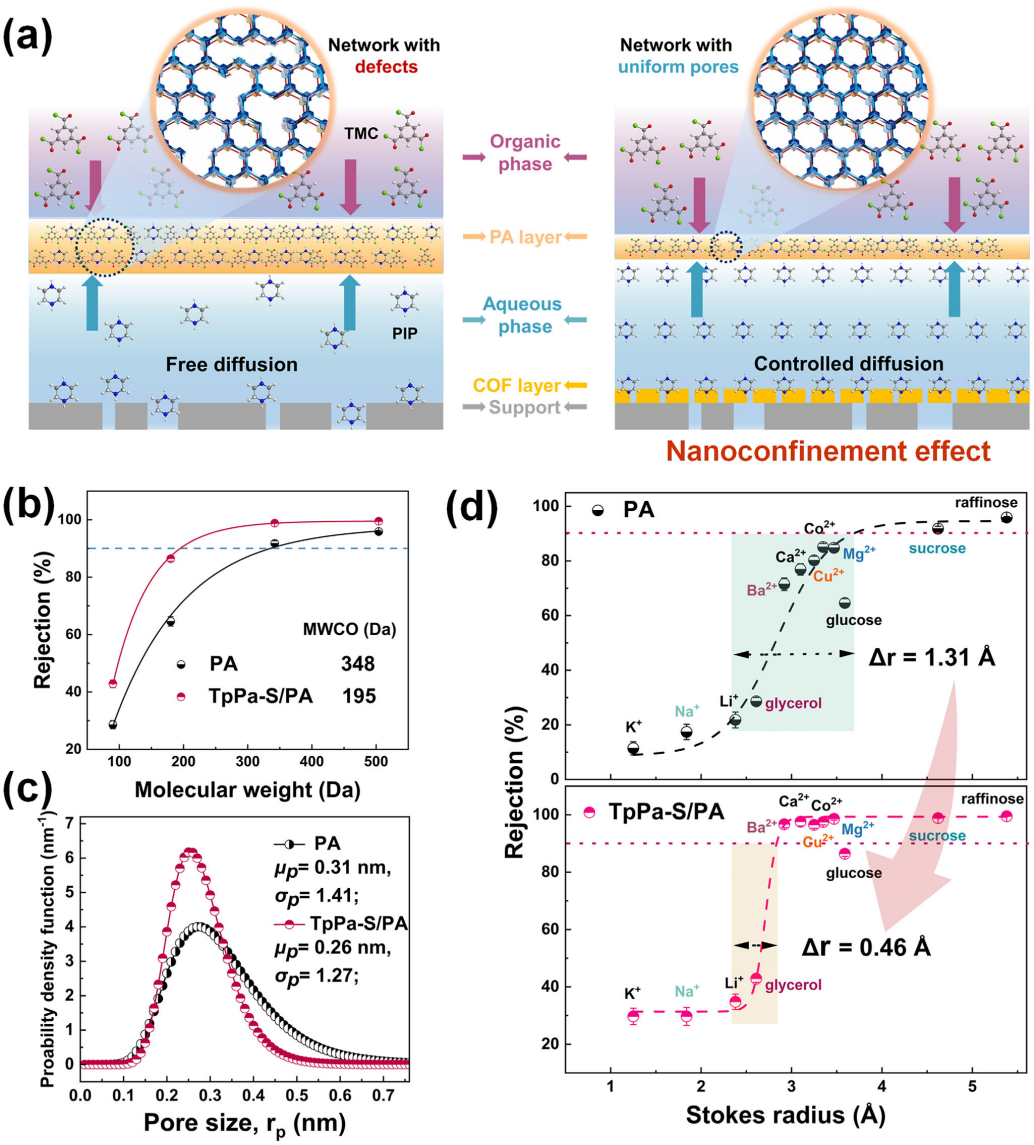
The sieving properties of PA layer with uniform pores modulated by COF nanoconfinement a) The mechanism of optimizing pore distribution via the TpPa‐S layer. b) MWCOs, c) pore size density distribution function, and d) rejection of different solutes as a function of the Stokes radius for the PA membranes with and without TpPa‐S nanofilm.

In theory, the formation of the PA selective layer during the IP process is a non‐equilibrium, self‐limiting diffusion reaction, primarily governed by the interfacial diffusion of the PIP monomer. As shown in **Figure** **3**a, in the conventional IP process, the hydrophobicity and uneven pore size of the PSF substrate prevent sufficient loading and uniform spreading of PIP monomers on its surface. When the PIP monomers diffuse to the water‐oil interface and react with TMC, this diffusion process is typically rapid and disorderly, resulting in a thicker selective layer with uneven pore size distribution. In contrast, the ordered nanoconfinement mechanism based on TpPa‐S layers demonstrates multiple advantages in the IP process. Due to their excellent hydrophilicity, TpPa‐S layers can enrich PIP monomers within their nanopores and on their surface, promoting a more uniform distribution during the reaction. This enrichment effect increases the concentration of PIP monomers when reacting with TMC, facilitating the formation of more cross‐linked structures and reducing defects in the PA layer. Additionally, the TpPa‐S layer, rich in secondary amine, carbonyl, and sulfonic acid groups, can form hydrogen bonds and electrostatic interactions with PIP monomers, as shown in Figure S16 (Supporting Information). Coupled with the physical confinement from the uniformly small pores, this further effectively regulates the diffusion behavior of PIP monomers, inducing a more ordered IP reaction. These results have been confirmed by simulations and experimental data. According to the Freeger equation,^[^ ^37^ ^]^ the enhanced monomer concentration and reduced diffusion rate effectively decrease the thickness of the PA layer. Through the ordered nanoconfinement effect of the TpPa‐S layer, not only is the spatial uniformity of PIP monomer distribution during diffusion significantly improved, but the polymerization reaction is also made more homogeneous. The resulting PA layer exhibits a more uniform pore size distribution while inhibiting further diffusion of PIP monomers, ultimately resulting in a thinner and more structurally uniform active selective layer.

To confirm the formation of a more uniform and narrower pore in PA layer, we selected several neutral polysaccharide molecules with varying molecular weights as probe molecules. As shown in Figure 3b,c, the MWCOs for PA and TpPa‐S/PA membranes are 348 and 195 Da, respectively, with corresponding mean effective radii of 0.31, and 0.26 nm. Compared to the pristine PA membrane, TpPa‐S/PA membranes exhibit narrower pore size distributions and smaller average pore sizes. Furthermore, the uniform pore structure of the NF membranes was evaluated using cations with varying hydrated ionic radii, as well as neutral molecules with different Stokes radii, to generate rejection curves. As shown in Figure 3d, Figure S17 and Table S4 (Supporting Information), the pristine PA membrane exhibited a broad rejection range (1.31 Å) for solutes with varying Stokes radii, indicating its limited ability to achieve high‐precision separation, especially for ions or molecules with similar sizes. Notably, the TpPa‐S layer‐modified PA membranes exhibited a much narrower transition region (0.46 Å) in the rejection curves, allowing these membranes to better differentiate between ions of similar sizes, particularly in the separation of Mg^2^⁺ and Li⁺. These results further confirm that the TpPa‐S layer could achieve the reconstruction of the PA layer with uniform and narrow channels for precise separation under the nanoconfinement manipulation mechanism.

**Figure 4:**
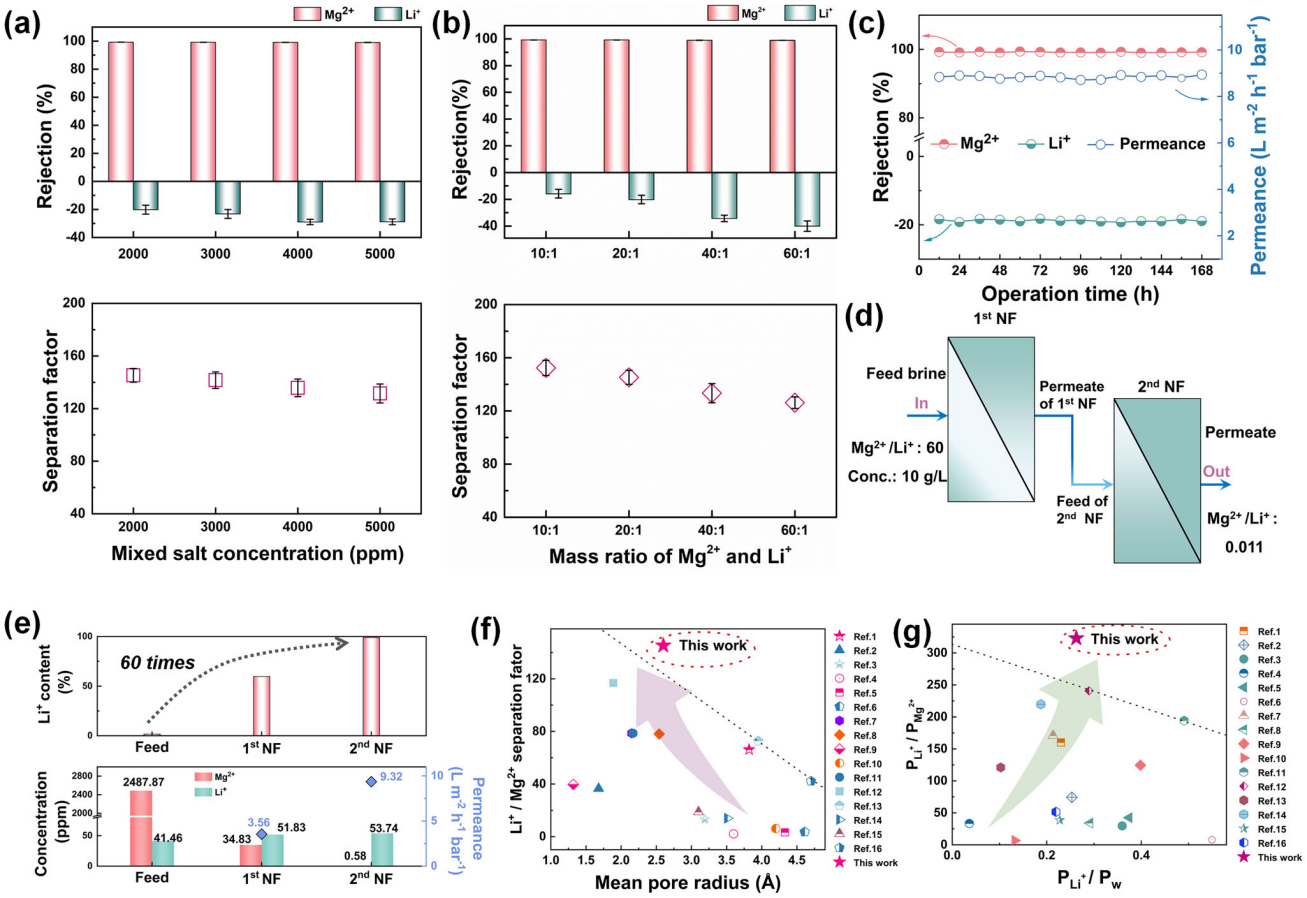
Li^+^/Mg^2+^ separation performance of TpPa‐S/PA membrane a) Variation in separation permeance as a function of the total concentration of mixed salts (Mg^2+^: Li⁺ = 20:1). b) Effect of the mass ratio of Mg^2+^ to Li⁺ on separation permeance, keeping the total mixed salt concentration fixed at 2 g L^−1^. c) Long‐term operational stability of the TpPa‐S/PA membrane in 2000 ppm simulated brine (1.866 g L^−1^ MgCl_2_, 0.134 g L^−1^ LiCl, Mg^2+^: Li⁺ = 20:1) under 10 bar pressure. d) Schematic representation of lithium enrichment via two‐stage filtration of simulated salt lake brine. e) Concentrations of Li⁺ and Mg^2+^ during the two‐stage filtration test (feed:2.48787 g L^−1^ Mg^2+^, 0.04146 g L^−1^ Li^+^); f) Separation factors of different membranes as a function of mean pore radius. g) Ratio of Li⁺ permeability to Mg^2+^ permeability (P_Li⁺_/P_Mg_^2+^) versus ratio of Li⁺ permeability to water permeability (P_Li⁺_/P_w_).

The precise separation ability of the designed membranes was systematically evaluated under cross‐flow conditions using a series of simulated single salt solution or mixed brine solution with different salt concentrations and Mg^2+^/Li^+^ mass ratios. As shown in **Figure** **4**a,b, and Figures S18–S20 (Supporting Information), under nanoconfinement effect regulation, both the TpPa/PA and TpPa‐S/PA membranes demonstrate excellent Li^+^/Mg^2+^ separation performance. This result highlights the crucial role of the COF layer in enhancing ion selectivity, further confirming its effectiveness in modulating the interfacial polymerization process and optimizing membrane structure for improved separation efficiency. Notably, it consistently maintains exceptional Li^+^/Mg^2+^ separation efficiency, regardless of variations in total concentration or mass ratio. The rejection rate for Mg^2+^ remains steadily above 98%, while the retention rate of Li^+^ is consistently below −14%, with the separation factor of Li^+^/Mg^2+^ reaching an impressive of 120. It is well‐recognized that ion rejection in mixed salt solutions is strongly influenced by the interactions between different ions in the system. Li⁺, with its smaller hydrated radius and lower binding energy compared to Mg^2^⁺, permeates more easily through the separation layer.^[^ ^39^ ^]^ In addition, the TpPa‐S/PA membrane exhibited remarkable stability over a continuous operation period of 168 h, as shown in Figure 4c. Despite minor fluctuations in flux and ion retention rates, the overall performance remained consistently stable, suggesting that the TpPa‐S/PA membrane is well‐suited for large‐scale industrial processes, particularly in Li extraction.

To achieve high‐purity Li separation and enrichment from feed solutions with high concentrations and elevated Mg^2+^/Li^+^ ratios, a two‐stage nanofiltration (NF) process was designed and implemented for Li^+^/Mg^2+^ separation (Figure 4d). The results shown in Figure 4e highlight the remarkable Li^+^/Mg^2+^ separation capability of the TpPa‐S/PA membrane, which remains highly effective even under extreme concentration and ratio conditions. Following the two‐stage NF process, the proportion of Li⁺ in the permeate was enhanced nearly 60‐fold compared to the feed solution, where the Mg^2+^ concentration in the permeate was dramatically reduced from the initial 2487 ppm to just 0.58 ppm, demonstrating effective Li enrichment. These results underscore the immense potential of the TpPa‐S/PA membrane for practical Li extraction, in challenging systems characterized by high concentrations and elevated Mg^2+^/Li^+^ ratio. This provides a robust foundation for its application in industrial‐scale separation processes.

Compared to other existing membranes, the TpPa‐S/PA separation layer has an average pore size precisely located between the Stokes radii (*r_s_*) of Mg^2+^ and Li^+^. Moreover, its pore size distribution is predominantly concentrated within the hydrated radius (*r_h_*) of Mg^2+^ and Li^+^, exhibiting significantly superior separation factors (Figure 4f; Table S5, Supporting Information). In practical applications, Li purity and recovery rate are the two core criteria for evaluating membrane separation performance. In this study, based on the STEM model,^[^ ^36^ ^]^ the ratio of Li^+^ permeation flux to Mg^2+^ permeation flux (P_Li⁺_/P_Mg_^2+^) was used to represent Li^+^ purity, while the ratio of Li^+^ permeation flux to water permeation flux (P_Li⁺_/P_w_) was used to represent Li recovery rate. As shown in Figure 4g and Table S5 (Supporting Information), commercial membranes typically achieve higher Li recovery rates but lower Li purity. In contrast, the TpPa‐S/PA membrane designed in this study achieves outstanding Li purity while maintaining a high recovery rate. Briefly, these findings demonstrate that designing and fabricating dense separation layers with sharpened pore size distribution is an effective strategy for enhancing membrane separation performance.

**Figure 5:**
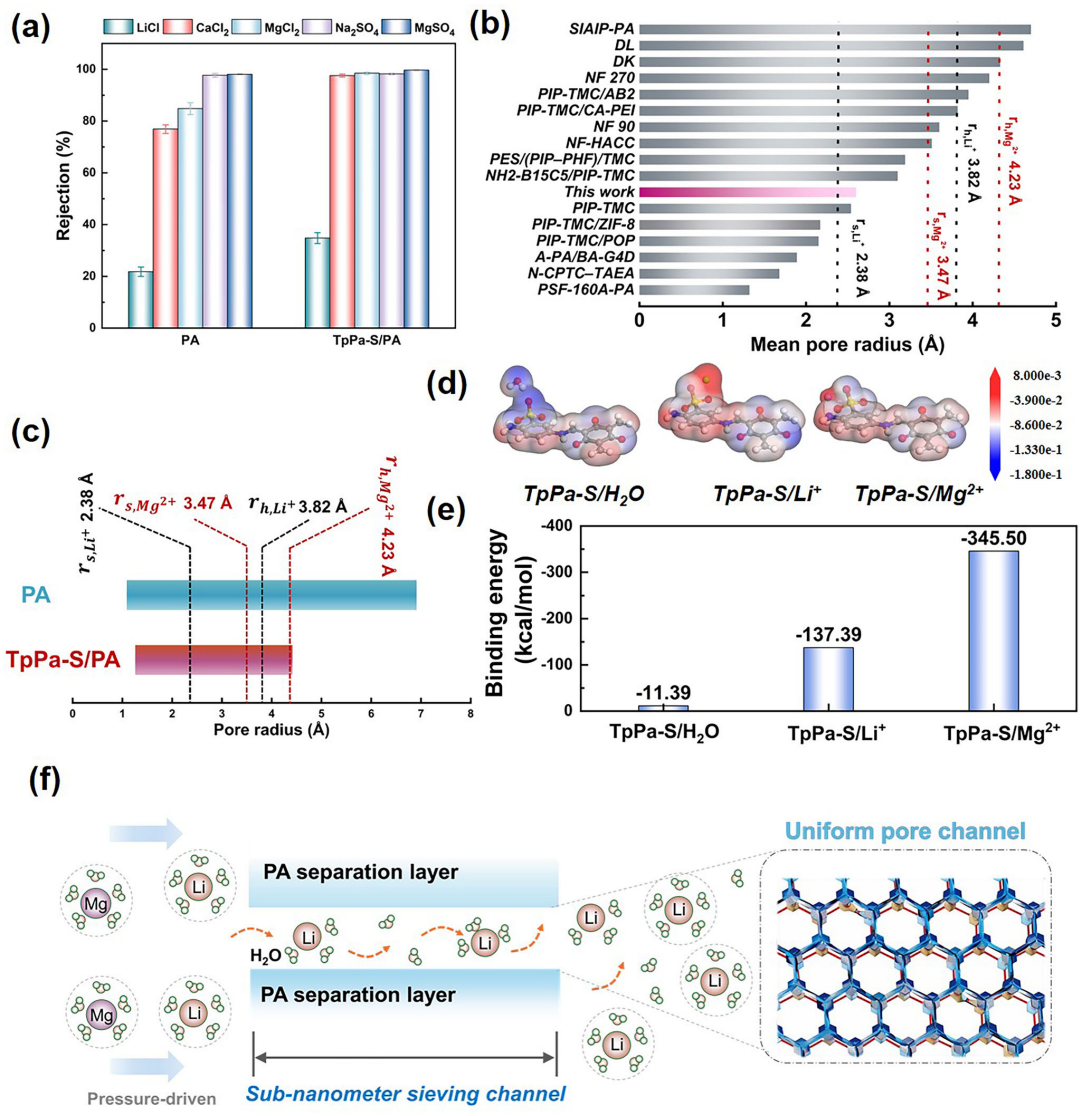
Li^+^/Mg^2+^ separation mechanism of TpPa‐S/PA membrane a) various salt rejection rates, b) mean pore size of membranes of this work and reported in other literature, c) comparison of pore size range of the pristine PA membrane and TpPa‐S/PA membrane, d) schematic diagram and e) results of MD simulation of binding energy between TpPa‐S units and H_2_O, Mg^2+^ and Li^+^, and f) schematic diagram of PA membrane under nanoconfinement regulation mechanism for precise separation of Mg^2+^ and Li^+^_._

Furthermore, although the TpPa‐S/PA membrane showed a negative charge (Figure S11, Supporting Information), it demonstrated an ultrahigh rejection rate (≈98%) for all divalent salts, including both anionic and cationic salts (**Figure** **5**a). This finding highlights the critical role of uniform pore channels in sieving ions, with minimal reliance on Donnan exclusion mechanisms. Compared with reported NF membranes, the TpPa‐S/PA membrane showed smaller effective pore channels and narrower pore size distribution, which is positioned between the Stokes radius of Mg^2+^and Li^+^ (Figure 5b,c), thereby allowing the separation layer to reject the majority of Mg^2+^ via size sieving while permitting the passage of most Li^+^. Furthermore, as hydrated ions transport across the confined channels, they have to undergo a dehydration process. Usually, Li⁺ possesses a significantly lower hydration energy (474 kJ mol⁻¹) compared to Mg^2+^ (1828 kJ mol⁻¹), making Li⁺ more readily dehydrated and facilitating its faster transport through the separation layer.^[^ ^40^, ^41^ ^]^ A sharp transition region is presented by the TpPa‐S/PA membrane when correlating the ion rejections with their stokes radius, suggesting its superior ion‐ion selectivity between the monovalent and divalent cations and more importantly the narrow, uniform pore channels of TpPa‐S/PA membrane is crucial for achieving superior Li^+^/Mg^2+^ separation.

Notably, the TpPa‐S/PA membranes also demonstrated substantial improvements in pure water permeance (7.3 L m^−2^ h^−1^ bar^−1^ for the pristine PA and 11.5 L m^−2^ h^−1^ bar^−1^ for TpPa‐S/PA), with the highest improvement reaching up to 1.6 times that of the pristine PA membrane (Figure S18, Supporting Information). This significant increase in permeability is mainly attributed to the reduction in PA layer thickness, which decreases mass transfer resistance and shortens the water transport pathways. It cannot be ignored that there are abundant sulfonic acid groups in the skeleton of TpPa‐S, which will be beneficial to the water transport and ion recognition process. As shown in Figure 5d,e, the MD simulation results revealed that the sulfonic groups in TpPa‐S skeleton exhibit a significantly higher binding energy with Mg^2+^ than with Li⁺, leading to stronger binding and reduced transport rates for Mg^2+^. Conversely, Li⁺, with weaker binding to sulfonic groups, passes through the TpPa‐S layer more rapidly. Specifically, based on the confinement effect of the hydrophilic charged TpPa‐S, the hydrogen bonding and electrostatic attraction between the oxygen atom on the sulfonic acid group and hydrated ions significantly restrict the behavior of Mg^2+^. In contrast, monovalent Li⁺, with lower charge density, can rapidly pass through the channels, thereby achieving exceptionally high separation performance.^[^ ^42^, ^43^ ^]^ Additionally, the presence of sulfonic acid groups in TpPa‐S alters the chemical microenvironment within the size‐constrained channels (Figure S21, Supporting Information), potentially increasing the difference in transmembrane activation free energy for Mg^2+^/Li⁺ and thus enhancing the membrane's ion recognition capability.^[^ ^44^ ^]^ Importantly, this significant disparity in ion transport rates further enhances the Li^+^/Mg^2+^ separation selectivity of the composite membrane. In addition, the sulfonic acid groups could also facilitate the more water molecules transport due to their well affinity, which is beneficial to the improvement of membrane permeability.^[^ ^45^, ^46^ ^]^ Therefore, under the manipulation of TpPa‐S COF nanoconfinement regulation mechanism, the PA layer could be finely adjusted to form a uniform and narrow pore structure (Figure 5f), and meanwhile, TpPa‐S layer endowed the superior ion recognition ability for whole membrane, which is crucial for achieving high‐performance Li^+^/Mg^2+^ separation process.

To further explore the regulatory role of TpPa‐S nanofilm in controlling the pore size and structure of separation layers, we employed a reverse interfacial polymerization (RIP) method to fabricate an alternative RTpPa‐S nanofilm, designed to modulate the IP process (Figure S22, Supporting Information). Similar to TpPa‐S layer, the formed RTpPa‐S layer showed the same chemical structure, which also decreased the MWCO of the substrate and provided a well nanoconfinement environment for following IP process (Figures S23 and S24, Supporting Information). Therefore, similar to TpPa‐S/PA, under the manipulation of RTpPa‐S, a uniform, and dense PA layer was also reconstructed, where the membrane thickness was also decreased and the chemical composition was adjusted to improve the membrane crosslinking degree (Figures S25–S27, Supporting Information). More importantly, the RTpPa‐S/PA membrane also showed a narrow pore size distribution (Figure S28, Supporting Information), which indicated that the TpPa‐S layer plays a crucial role of achieving the formation of uniform pore channels in the PA membrane and the PA layer was intensively correlated to the TpPa‐S nanoconfinement regulation mechanism rather than the growth style. Correspondingly, the RTpPa‐S/PA membrane also showed an improvement of Li^+^/Mg^2+^ separation performance (Figures S29 and S30, Supporting Information), which further confirmed our nanoconfinement regulation strategy is successful in achieving the reconstruction PA layer with uniform pores and well‐pore chemical microenvironment.

## 3 Conclusion

In conclusion, we proposed a nanoconfinement regulation strategy to construct a dense PA separation membrane with uniformly tailored pores, achieving highly selective separation of Li^+^ over Mg^2+^. This strategy employed a TpPa‐S COF rich in abundant sulfonic acid side chains, which created a well‐nanoconfined microenvironment. The TpPa‐S layer effectively regulated the diffusion of PIP monomers and enriched their concentration near the membrane surface aiming to manipulate the interfacial chemistry reaction process for IP. The nanoconfinement strategy successfully reconstructed both the pore structure and the chemical composition of the PA layer. As‐obtained TpPa‐S/PA membrane showed a uniform and narrow pore environment, which exhibited exceptional Li^+^/Mg^2+^ separation performance, achieving a separation factor exceeding 120. In addition, a two‐stage filtration process of the simulated salt lake brine equipped with TpPa‐S/PA membrane further demonstrated its high potential for practical lithium extraction. More importantly, comprehensive investigations into ion transport mechanisms revealed that size sieving played a crucial role in enhancing separation performance. This study highlights the nanoconfinement regulation mechanism for interfacial chemistry in advancing the design of selective ion separation membranes, establishing a promising pathway for scalable and efficient Li extraction technologies.

## Conflict of Interest

The authors declare no conflict of interest.

## Author Contributions

S.Z. conceptualized the research, performed methodology development, and investigation, and wrote as well as reviewed the manuscript. L.D. contributed to methodology, validation, supervision, and manuscript review. Z.M. was responsible for software development, methodology, and simulation. B.L., A.M., and P.Z. conducted investigations. M.Z. contributed to investigation and software development. K.G. and R.T. supervised the research, validated data, and curated data. H.M. was responsible for review and editing, supervision, funding acquisition, and methodology. All authors contributed to the article and approved the submitted version.

## Supporting information



Supporting Information

## Data Availability

The data that support the findings of this study are available from the corresponding author upon reasonable request.
