# Supplementary material for: Nanoconfinement Engineering of Covalent Organic Frameworks in Polyamide Membranes for High‐Perselectivity Li+/Mg2+ Separation
Source: Adv Sci (Weinh). 2025 Apr 2;12(23):2500255. doi: 10.1002/advs.202500255 (PMC12199429; doi:10.1002/advs.202500255)
Supplement: Supplementary file 1 — Supporting Information [file ADVS-12-2500255-s001.docx]

***Supporting Information***

**Nanoconfinement Engineering of Covalent Organic Frameworks in Polyamide Membranes for High-Perselectivity** **Li^+^/Mg^2+^ Separation**

Shuzhen Zhao ^a,b^, Liheng Dai *^a^, Zhaohuan Mai ^a^, Bowen Li ^a,b^, Pengfei Zhang ^a^, Mengxiao Zhang ^c^, Atsushi Matsuoka ^a^, Kecheng Guan ^a^, Ryosuke Takagi ^a^, Hideto Matsuyama *^a,b^

*^a^ Research Center for Membrane and Film Technology, Kobe University, 1-1 Rokkodaicho, Nada, Kobe, 657-8501, Japan*

*^b^ Department of Chemical Science and Engineering, Kobe University, 1-1 Rokkodaicho, Nada, Kobe, 657-8501, Japan*

*c MOE Key Laboratory of Macromolecular Synthesis and Functionalization, Department of Polymer Science and Engineering, Zhejiang University, Hangzhou 310027, China*

** Corresponding author, email: lhengdai@people.kobe-u.ac.jp and matuyama@kobe-u.ac.jp*

1. **Experimental**
   1. **Materials**

Commercial PSF membranes (US-050) were purchased from RisingSun Membrane Technology (Beijing) Co., Ltd and used as the substrates for the IP process. 2,4,6-triformylphloroglucinol (Tp), p-phenylenediamine (Pa), 2,5-diaminobenzenesulfonic acid (Pa-SO_3_H) were obtained from Fluorochem Ltd. 1, 3, 5, -benzentricarbonyl (TMC), acetic acid, n-hexane, sodium chloride (NaCl), potassium chloride (KCl), lithium chloride (LiCl), magnesium chloride (MgCl_2_), calcium chloride (CaCl_2_), copper (II) chloride (CuCl_2_), cobalt chloride (CoCl_2_), barium chloride (BaCl_2_), magnesium sulfate (MgSO_4_) and sodium sulfate (Na_2_SO_4_) were purchased from FUJIFILM Business Innovation Co. Anhydrous piperazine (PIP), dextrans with different molecular weights (*M_w_* = 20 kDa, 60 kDa, 100 kDa, 150 kDa, 200 kDa) , glycerol (*M_w_* = 92.1 Da), D-(+)-glucose (*M_w_* = 180.2 Da), sucrose (*M_w_* = 342.3 Da), and D(*+*)-raffinose pentahydrate (*M_w_* = 594.5 Da) were obtained from Sigma-Aldrich.

- 1. **Preparation of TpPa and TpPa-S COF layers**

The COF layers were fabricated via *in-situ* interfacial polymerization between Pa or Pa-SO_3_H and Tp (**Fig.S1**). The detailed preparation process is as follows. The preparation of the COF interlayer through *in-situ* interfacial polymerization closely resembles the conventional interfacial polymerization method. First, a 0.4 g/L aqueous solution of Pa-SO_3_H or Pa was poured onto the pre-fixed PSF substrate for 5 minutes, followed by removing the excess aqueous phase from the surface of the substrate using an air knife. Subsequently, a 0.04 g/L hexane solution of Tp was poured onto the PSF substrate to initiate in-situ interfacial polymerization for 5 minutes, yielding the PSF membrane modified by the COF layers. TpPa and TpPa-SO_3_H layer modied PSF substrates, were denoted as TpPa/PSF and TpPa-S/PSF, respectively.


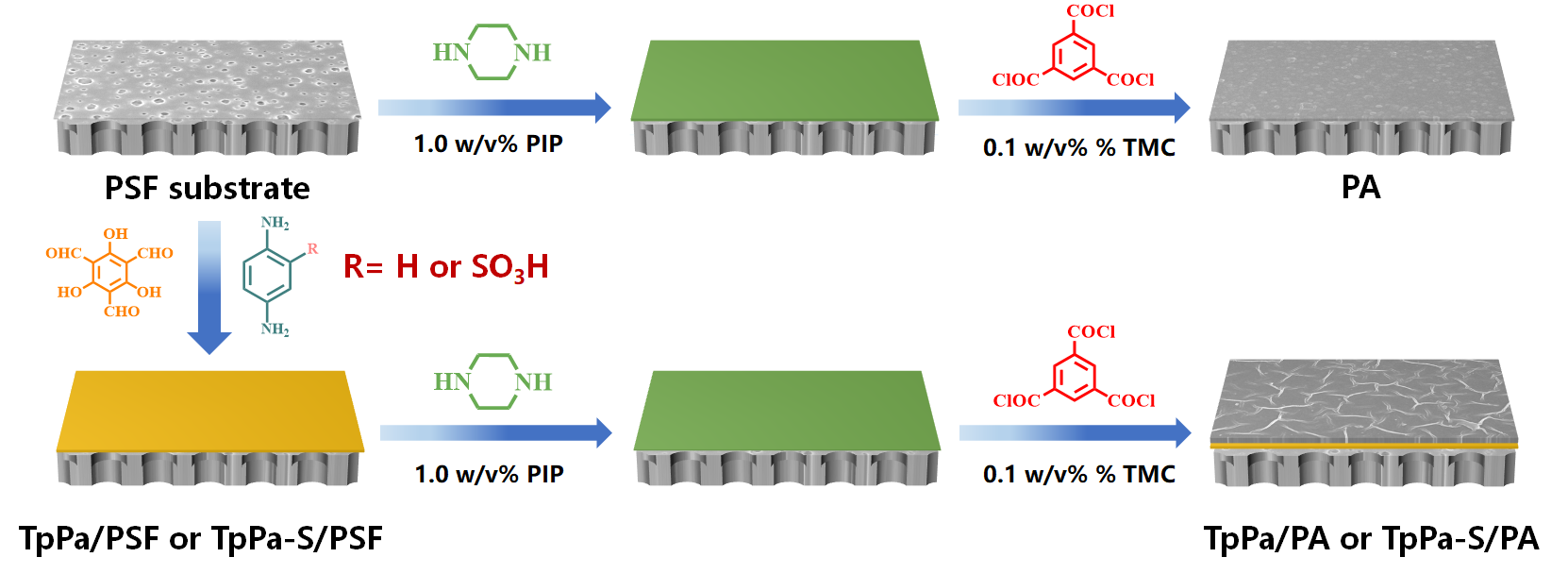


**Fig.S1** Schematic of TpPa or TpPa-S interlayers and TpPa or TpPa-S layer -modulated PA nanofiltration membrane preparation.

- 1. **Preparation of COF layer-modulated PA NF membranes**

As depicted in **Fig.S1**, the NF membranes were fabricated through the modulation of the TpPa and TpPa-S COF layers. The process began by applying a 1 w/v% aqueous solution of PIP onto the substrate surface for 2 minutes. Excess solution was removed using an air knife until no visible droplets remained on the surface. Subsequently, a 0.1 w/v% solution of TMC in n-hexane was introduced to initiate interfacial polymerization with the PIP monomer for 30 seconds, resulting in the formation of the PA active layer. The membrane surface was then rinsed with n-hexane to remove any unreacted monomers, followed by heating in an oven at 60°C for 3 minutes.

Adopting a similar naming convention as for the COF layers, the PA membranes fabricated on the pristine PSF substrate, TpPa/PSF substrate and TpPa-S/PSF substrate are designated as PA, TpPa/PA and TpPa-S/PA membranes, respectively.

- 1. **Characterization**

The chemical composition and surface properties of the membranes were analyzed using attenuated total reflection Fourier-transform infrared spectroscopy (ATR-FTIR, PerkinElmer) and X-ray photoelectron spectroscopy (XPS, JPS-9010 MC, JEOL). The hydrophilicity of the membrane surface was assessed by measuring the water contact angle (WCA, Drop Master 300, Kyowa) with a goniometer. To evaluate the surface charge characteristics, the zeta potential was determined using a solid surface analyzer (Anton paar, Australia). The surface morphology, structure, and thickness of the active layer were examined via field emission scanning electron microscopy (FESEM; Sigma 500, ZEISS). Atomic force microscopy (AFM, Bruker, Dimension Icon) was employed to evaluate surface roughness and morphology. Additionally, a UV–Vis spectrophotometer (SHIMADZU) was used to determine solute concentrations in hexane. Inductively Coupled Plasma Mass Spectrometry (ICP-MS, SHIMADZU) was employed to measure the ion concentrations in the mixed salt solution.

Using the established neutral solute transport model, 0.2 g.L^-1^ four polysaccharides and five dextran molecules with varying molecular weights were employed to separately determine the molecular weight cut-off (MWCO) and pore size distribution density function of the substrates and PA separation layers, respectively. The neutral solutes transport method was employed to determine the pore size and pore size distribution of the membranes. Aqueous solutions of dextrans (200 ppm) and raffinose, sucrose, glucose, and glycerol (1000 ppm) were used to assess the pore properties of PSF ultrafiltration supports and PA-based membranes, respectively. The rejection ratios were measured using a total organic carbon analyzer (TOC, TOC-V, SHIMADZU) and calculated Membrane pore size distribution curves were then derived using the following equation:

$$\frac{dR(d_{p})}{dd_{p}}=\frac{1}{d_{p} ln\sigma_{p}\sqrt{2\pi}}exp\left[ -\frac{\left( {lnd}_{p}-ln\mu_{p} \right)^{2}}{2\left( ln\sigma_{p} \right)^{2}} \right] (S1)$$

where the membrane pore size (*μ_p_*, nm) is the same as the solute size (*r_p_*, nm) at a rejection rate of 50%; the Stokes radius (*r_s_*, nm) equals the solute size at a rejection rate of 84.13%, and the geometric standard (*σ_p_*) is calculated as the ratio of ds to *d_p_*.

The Stokes radii (*r_p_*, nm) of these neutral organic compounds were calculated using the following equations.

For small neutral carbohydrate molecules:

$$log\left( r_{p} \right)=-1.4962+0.4654logM_{w} (S2)$$

For Dextrans molecules:

$$r_{p}=10.44\times{{{10}^{-3}M}_{W}}^{0.587} (S3)$$

Based on the above analysis, the Stokes radius of glycerol, glucose, sucrose, and raffinose were 0.26, 0.36, 0.48, and 0.58 nm, respectively.

- 1. **Molecular Dynamics (MD) simulations**

The underlying mechanism responsible for the reduced diffusion rate of PIP into the organic phase, due to the presence of TpPa-S COF layers, was investigated using the Forcite module in Materials Studio 2023, applying the COMPASS II force field. A cubic simulation box with periodic boundary conditions in all three directions was established to model the PIP solution (1.0 w/v%). To simulate the influence of the TpPa-S COF layer, 1 repeating unit of TpPa-S COF was introduced into the cubic box representing the TpPa-S/PIP system. Although this model was simplified, the molecular dynamics (MD) simulation quantitatively demonstrated that the TpPa-S COF layer significantly hinders the diffusion of PIP. Geometric optimization was first performed for both the PIP and TpPa-S/PIP systems, followed by MD simulations for 0.5 ns with a 1 fs time step, using a constant number of particles, volume, and temperature (NVT) ensemble. The initial velocities of water, PIP, and SCOF molecules were randomized, and the system temperature was maintained at 298.0 K using a Nose thermostat. Subsequently, an additional 0.5 ns simulation was conducted under constant particle number, pressure, and temperature (NPT) conditions, followed by a 5 ns simulation using the NVT ensemble. After reaching equilibrium with respect to energy and temperature, data from the final 5 ns of the NVT simulation were collected to analyze diffusion properties. The diffusion coefficients of PIP were then calculated based on Einstein’s equation.

| $MSD=\frac{1}{N}\sum_{i=1}^{N} \left\langle\left[ r_{i}\left( t+t_{0} \right)-r_{i}\left( t_{0} \right) \right]^{2} \right\rangle=A+6D_{a}t$ | (*S4*) |
| --- | --- |

where mean square displacement (MSD) was calculated for the center of gravity of the PIP in the water system. The diffusion coefficient was derived by analyzing the slope of the MSD as a function of time within a specific time range^[1]^.

- 1. **Determination of COF layers on PIP diffusion rates into n-hexane**

To evaluate the diffusion behavior of PIP monomers into n-hexane, a procedure analogous to the interfacial polymerization (IP) process was utilized. Initially, 25 ml of aqueous PIP solution was applied to the surface of pristine PSF, TpPa/PSF and TpPa-S/PSF substrates for 2 minutes. Excess solution was then removed using an air knife. The substrates were subsequently immersed in 20 ml of pure n-hexane (without TMC) for 30 seconds. After this, 1 ml of a hexane solution containing PIP monomer was carefully pipetted at the hexane-air interface. The concentration of PIP in the n-hexane phase was measured using UV–Vis absorption spectroscopy, providing a basis for calculating the diffusion rate of PIP into n-hexane.

- 1. **Effects of COF layers on PIP storage capacity**

The pristine PSF, TpPa/PSF and TpPa-S/PSF substrates, each with an effective surface area of 20.25 cm², were first immersed in 30 ml of an aqueous PIP solution (1 w/v%) for 2 minutes. Afterward, any excess liquid was removed from the substrates using an air knife. The substrates were then transferred to 40 ml of n-hexane without TMC, where they remained until the PIP monomers had fully diffused. The amount of PIP absorbed onto the substrate was quantified using UV–Vis absorption spectroscopy, with PIP showing a characteristic absorption peak near 198 nm, which corresponds to its concentration in n-hexane.

- 1. **Separation performance test of PA NF membranes**

The inorganic salt rejection (R) and permeance (J) of the NF membranes were evaluated using a cross-flow filtration system with an effective filtration area of 20.42 cm², and all of concentration of salt solution was 2 g L^-1^.

The water permeance (*J*), salt rejection (*R*), and single salt LiCl/MgCl_2_ selectivity ${(S}_{LiCl/{MgCl}_{2}}\boldsymbol{)}$were calculated by following equations

$J=\frac{\Delta V}{\Delta P\times\Delta t\times S}$ (*S5*)

$R=\left( 1-\frac{C_{p}}{C_{f}} \right)\times100\%$ (*S6*)

$S_{LiCl/{MgCl}_{2}}=\frac{1-R_{LiCl}}{1-R_{{MgCl}_{2}}}$ (*S7*)

where ΔV (L) represents the volume of permeated water over a time interval Δt (h), and ΔP is the transmembrane pressure. A transmembrane pressure of 10 bar and 2 bar was applied to evaluate the permeance of the PA-based membranes and PSF ultrafiltration supports, respectively. S (m²) denotes the effective filtration area. All membrane samples were pre-compacted for 2 hours until a stable water permeance was achieved prior to data collection. The reported water permeance values were calculated as the average of three measurements for samples prepared in different batches. Where C_p_ ​ and C_f_ represent the concentrations of the permeate and feed solutions, respectively. The rejection rates, *RLiCl* and *RMgCl_2_* correspond to the rejection of LiCl and MgCl_2_ solutions, respectively.

To further investigate the separation selectivity of membrane for mixed salt solution (MgCl_2_/LiCl), the separation factor ($S_{{{Li}^{+}}/{{Mg}^{2+}}}$) was also calculated according to this Eq.:

$S_{{{Li}^{+}}/{{Mg}^{2+}}}=\frac{\left( {C_{{f Mg}^{2+}}}/{C_{{f Li}^{+}}} \right)}{\left( {C_{{p Mg}^{2+}}}/{C_{{p Li}^{+}}} \right)}=\frac{1-R_{{Li}^{+}}}{1-R_{{Mg}^{2+}}}$ (S8)

here, $C_{{f Mg}^{2+}}$, $C_{{f Li}^{+}}$, $C_{{p Mg}^{2+}}$ and$C_{{p Li}^{+}}$ represent the concentrations of Mg²⁺ and Li⁺ in the feed and permeate, respectively. Each membrane sample was tested at least three times, with three separate membranes used for reproducibility.

We evaluated lithium recovery using the ratio of lithium permeability to water permeability (P_Li_/P_w_).

$LiR\equiv\frac{P_{Li}}{P_{w}}$ (S9)

where $P_{Li}$ and $P_{w}$ are lithium permeability to water permeability respectively.

For single-component solutions containing only Mg^2+^ or Li⁺, the ion concentrations were determined using an ion conductivity meter (Horiba, Japan). For the Mg²⁺/Li⁺ mixed solutions, the concentrations of Li⁺ and Mg^2^⁺ were measured using an inductively coupled plasma optical emission spectrometer.

1. **Results**


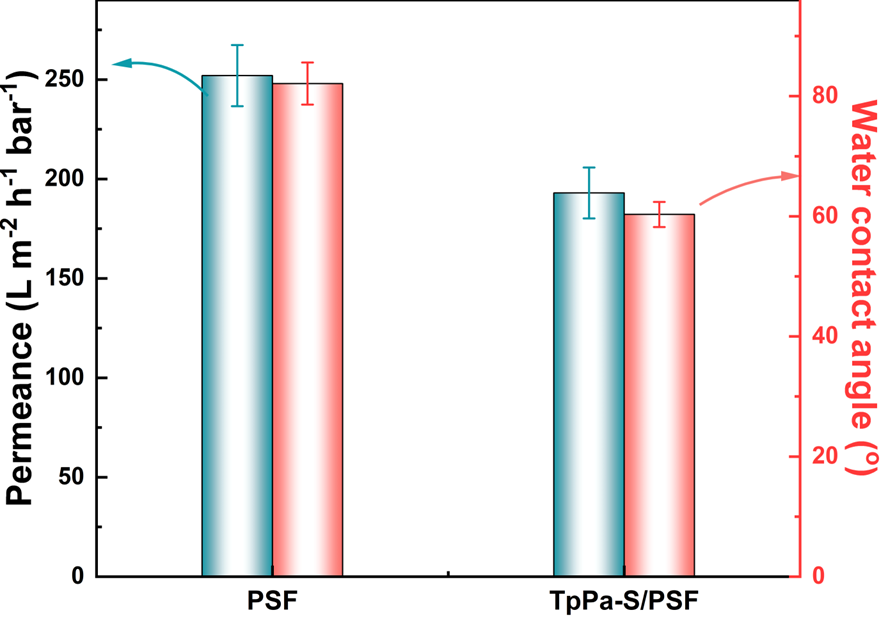


**Fig.S2** Pure water permeance and water contact angle of the PSF substrates with and without the TpPa-S COF layers.


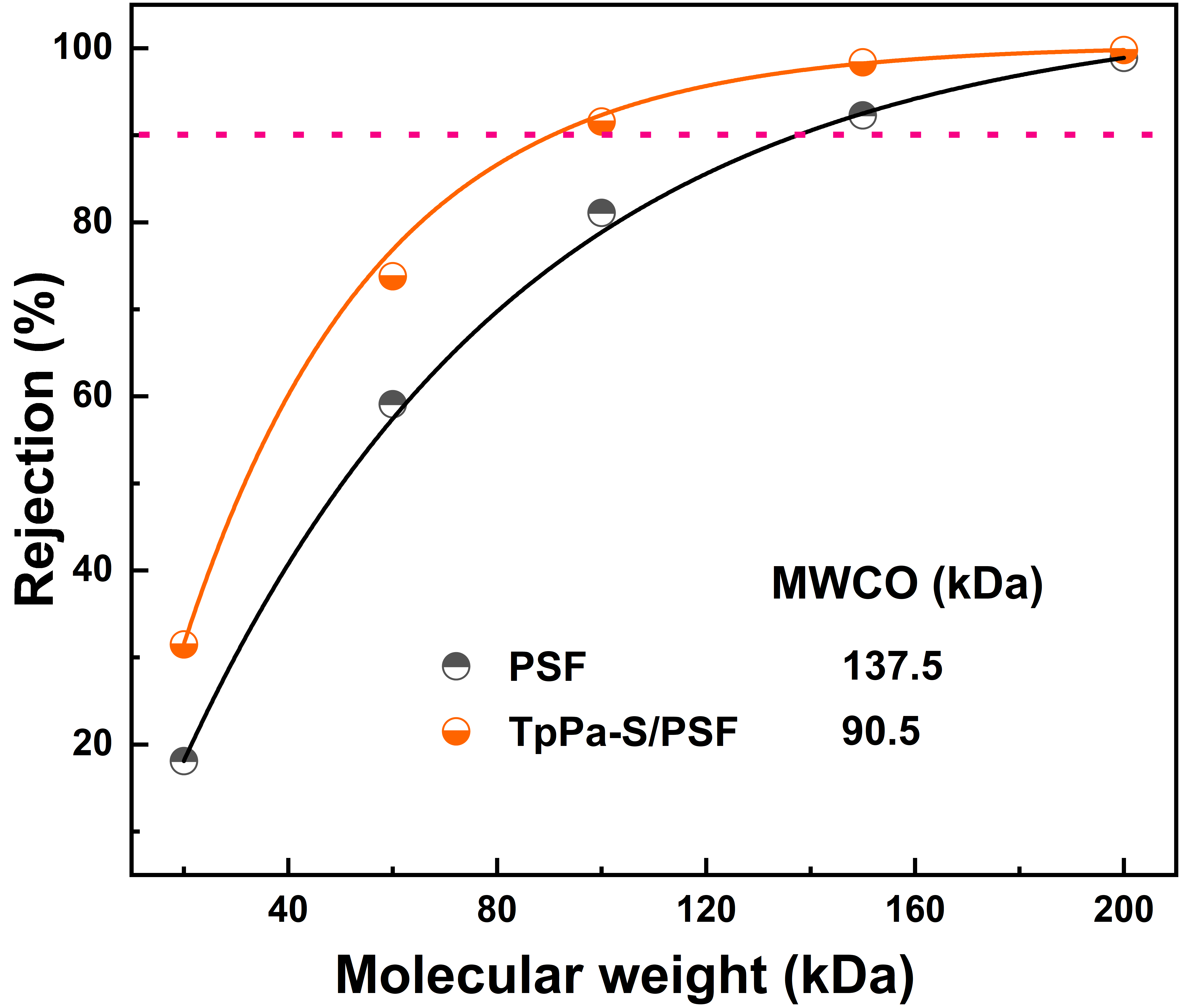


**Fig.S3** Surface morphology of the PSF substrates with and without the TpPa-S COF layers.


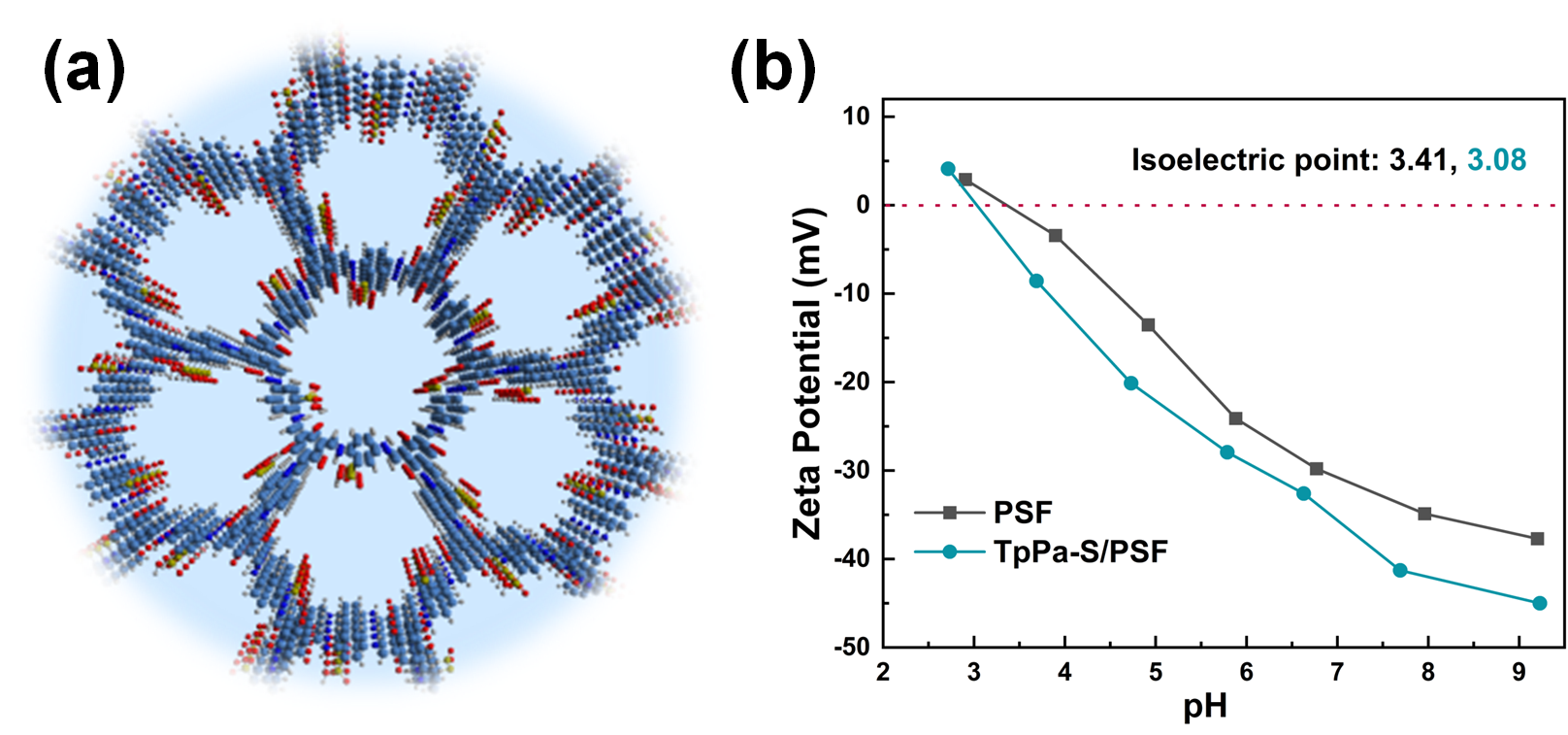


**Fig.S4** (a) **S**COF structure and (b) surface zeta potential of the PSF substrates with and without the TpPa-S COF layers.


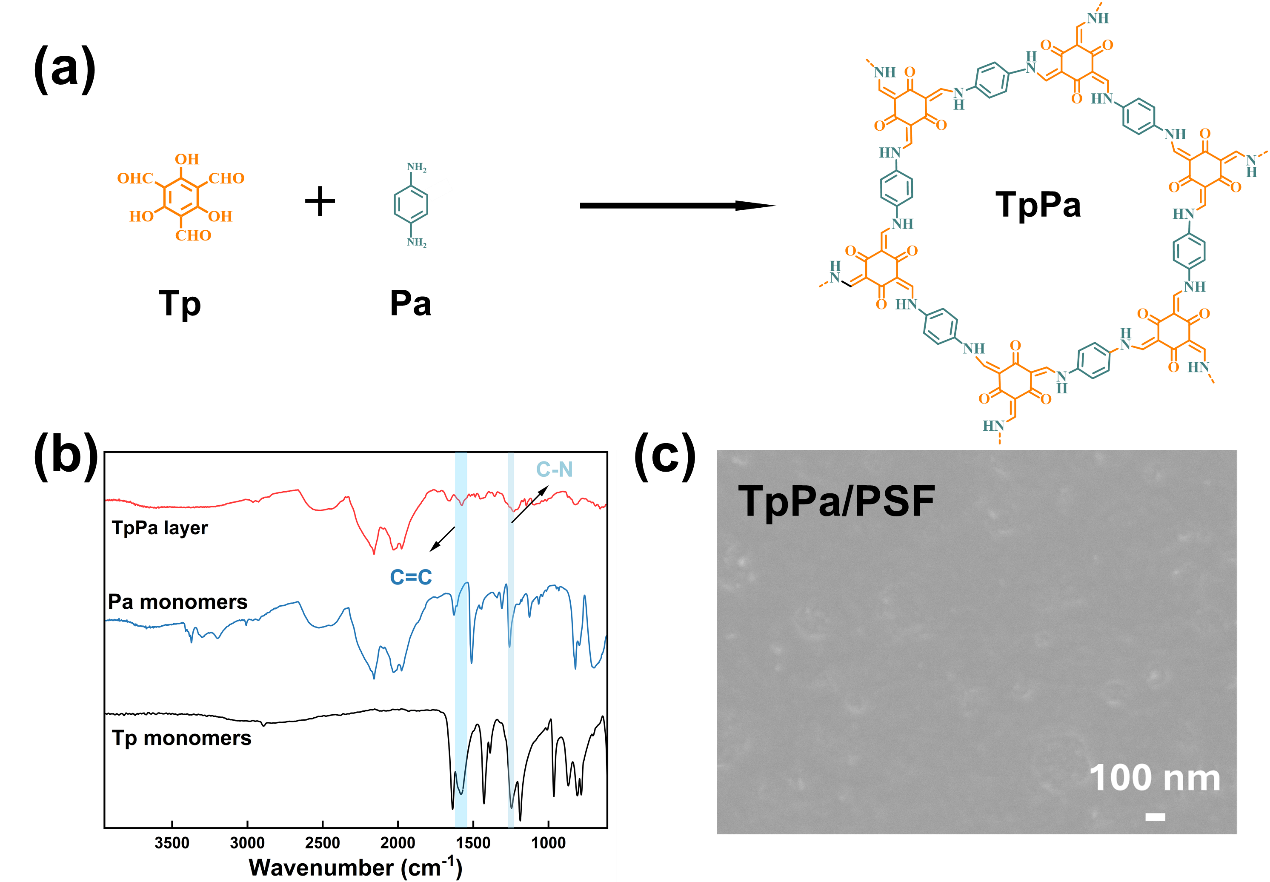


**Fig.S5** (a) Preparation route, (b) ATR-FTIR and (c) surface morphology of the TpPa COF layer


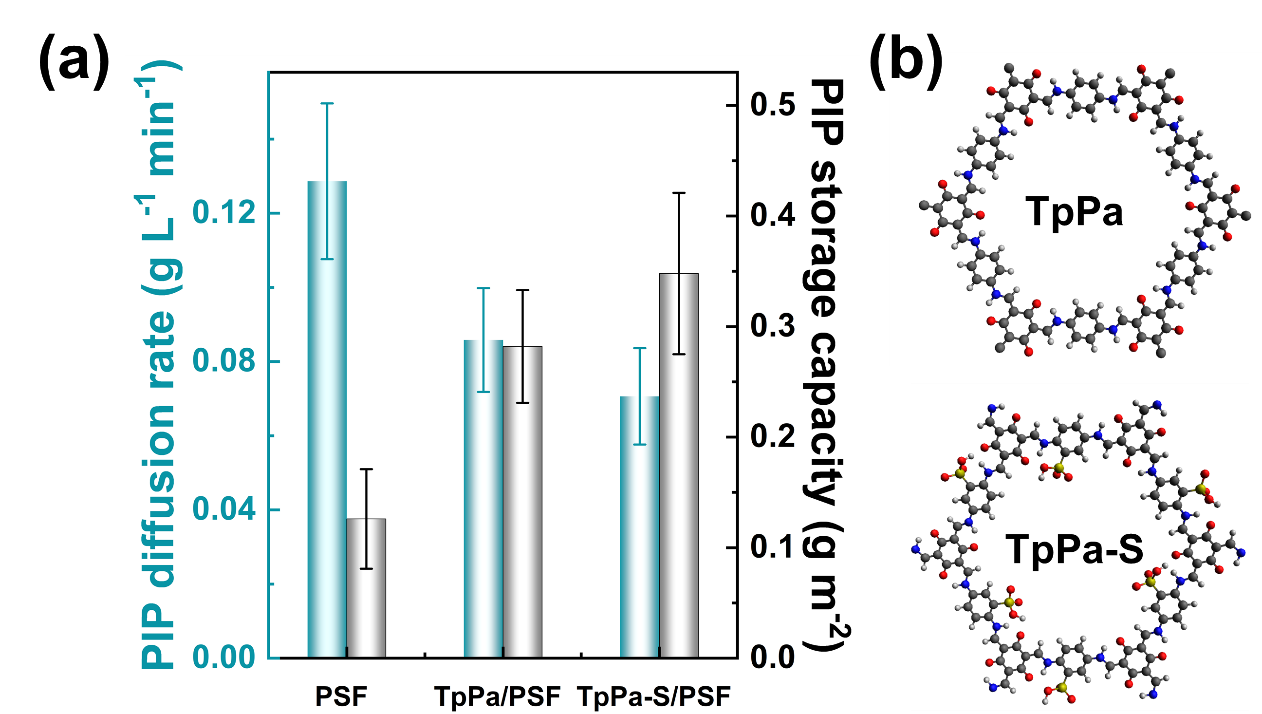


**Fig.S6** (a) The diffusion rates of PIP into n-hexane and PIP adsorption capacity on PSF substrates with and without COF nanofilm and (b) Ball-and-stick models structure of TpPa and TpPa-S COF


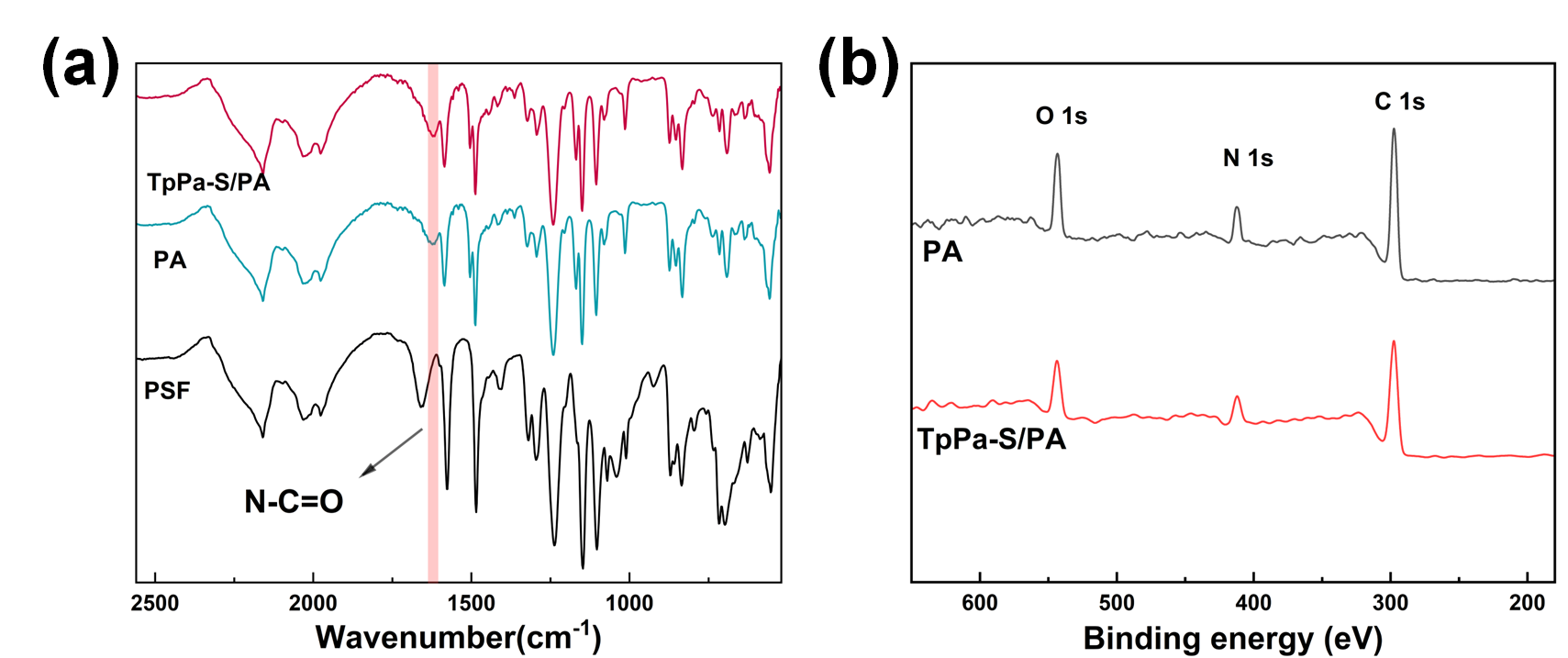


**Fig.S7** (a) The ATR-FTIR and (b) XPS spectra of the prepared NF membranes.

**Table S1 Elemental mass ratio of the membrane**

| **Membranes** | **C (%)** | **N (%)** | **O (%)** | **S (%)** |
| --- | --- | --- | --- | --- |
| PSF | 83.07 | Nd | 8.92 | 8.01 |
| TpPa-S/PSF | 69.58 | 9.12 | 8.96 | 12.34 |
| TpPa-S/PA | 60.41 | 18.83 | 20.25 | 0.01 |

To further validate the relationship between TpPa-S COF layer and PA layer, EDS mapping was performed to analyze the elemental composition of the PSF substrate, TpPa-S/PSF, and TpPa-S/PA surfaces, as shown in Fig. S8 and Table S1 Compared to the PSF membrane, the TpPa-S/PSF surface exhibits significant changes in elemental composition, most notably the appearance of nitrogen (N), which confirms the successful construction of the TpPa-S COF layer on the PSF substrate. Moreover, after interfacial polymerization, the surface elemental composition undergoes further transformation, with sulfur (S) becoming nearly undetectable. This provides strong evidence that the PA layer has completely covered the COF layer, further supporting the successful fabrication of the composite membrane.


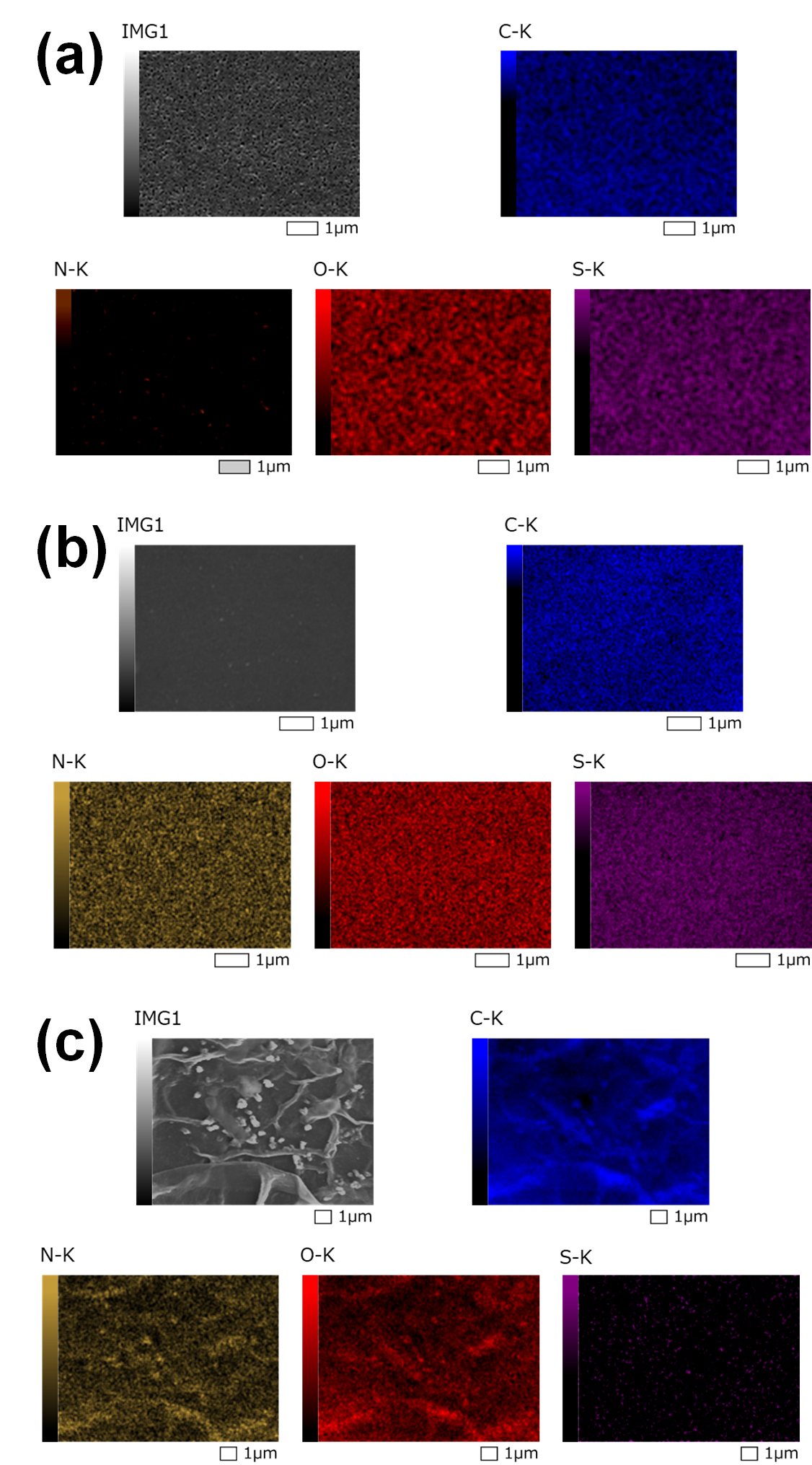


**Fig.S8** EDS surface images of (a) PSF, (b) TpPa-S/PSF and (c) TpPa-S/ PA membranes


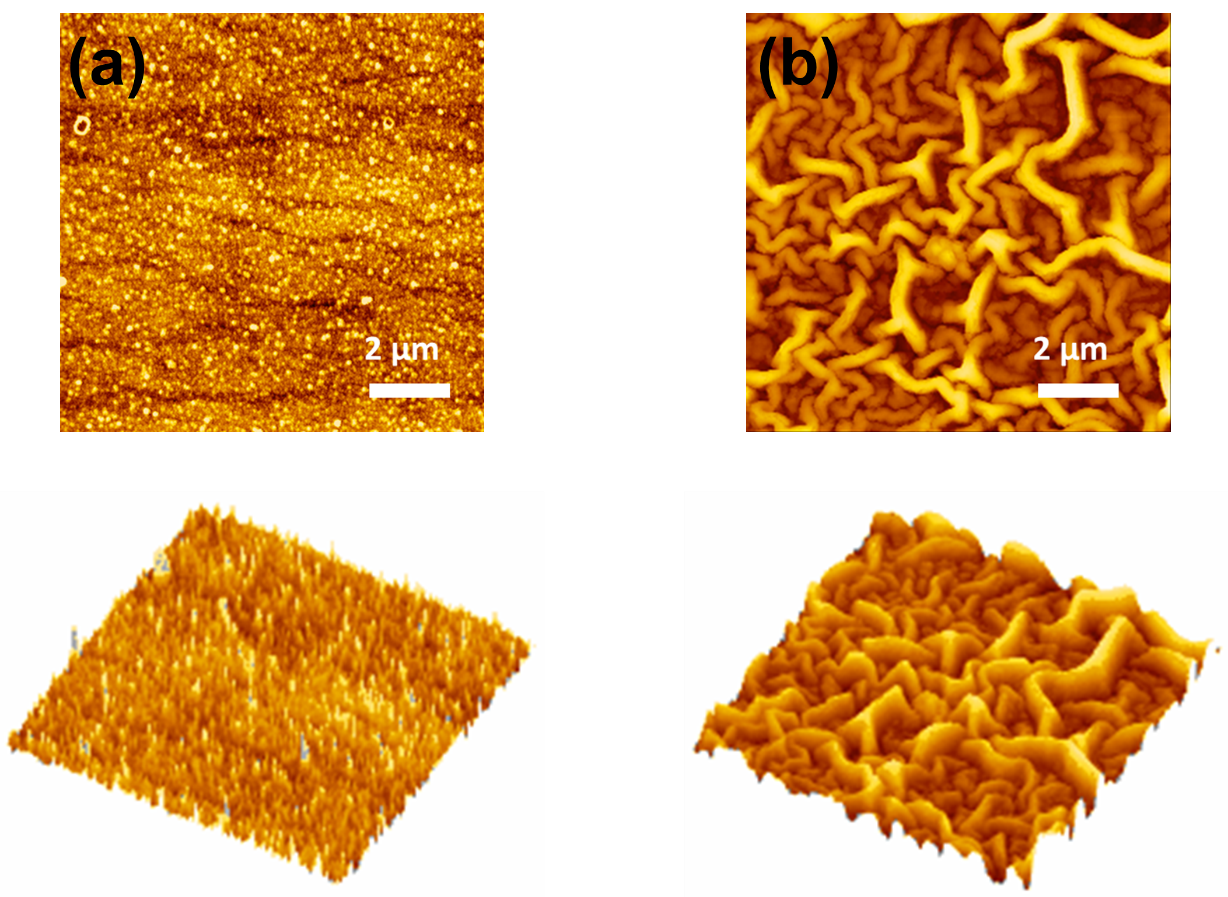


**Fig.S9** AFM images and 3D images of (a) PA, (b) TpPa-S/PSF PA

**Table S2** The roughness and thickness of the NF membranes.

| Membranes | Roughness (nm) | | Thickness of PA layer (nm) |
| --- | --- | --- | --- |
|  | Ra | Rms |  |
| PA | 12.3 | 16.8 | 83.7 |
| TpPa-S/PA | 65.9 | 80.4 | 54.2 |


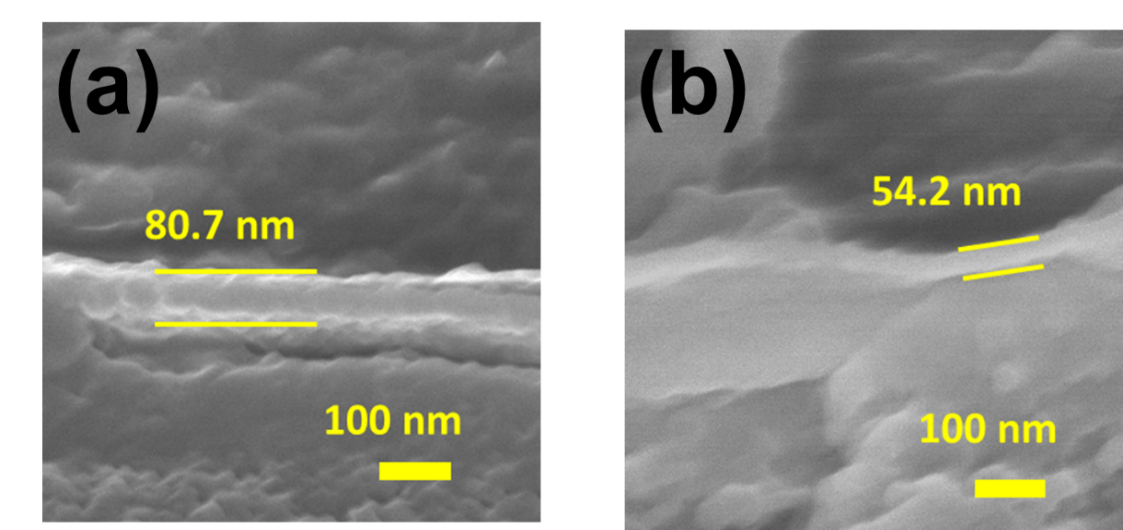


**Fig.S10** SEM cross-section images of (a) PA and (b) TpPa-S/PA


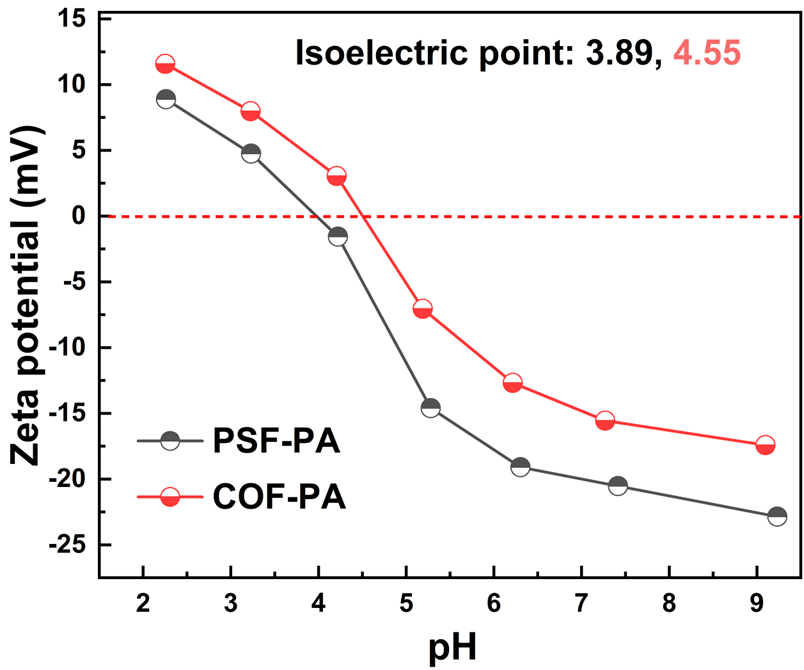


**Fig.S11** Surface zeta potential of the PA and TpPa-S/PA membranes.

**Cross-linking degree of PA membranes**

The degree of crosslinking (DC) in PA-based Thin Film Composite (TFC) membranes was determined by analyzing the atomic concentrations of elements within the membrane. This analysis is essential for understanding the membrane's structural properties and was performed using the following formula:

$$DC=\frac{X}{X+Y}\times100\% (S4)$$

where X represents the proportion of crosslinked structures, and Y represents the proportion of linear structures. where X and Y can be calculated based on the O and N contents using the following equations:

$$X+Y=1 (S5)$$

$$\frac{O}{N}=\frac{3X+4Y}{3X+2Y} (S6)$$

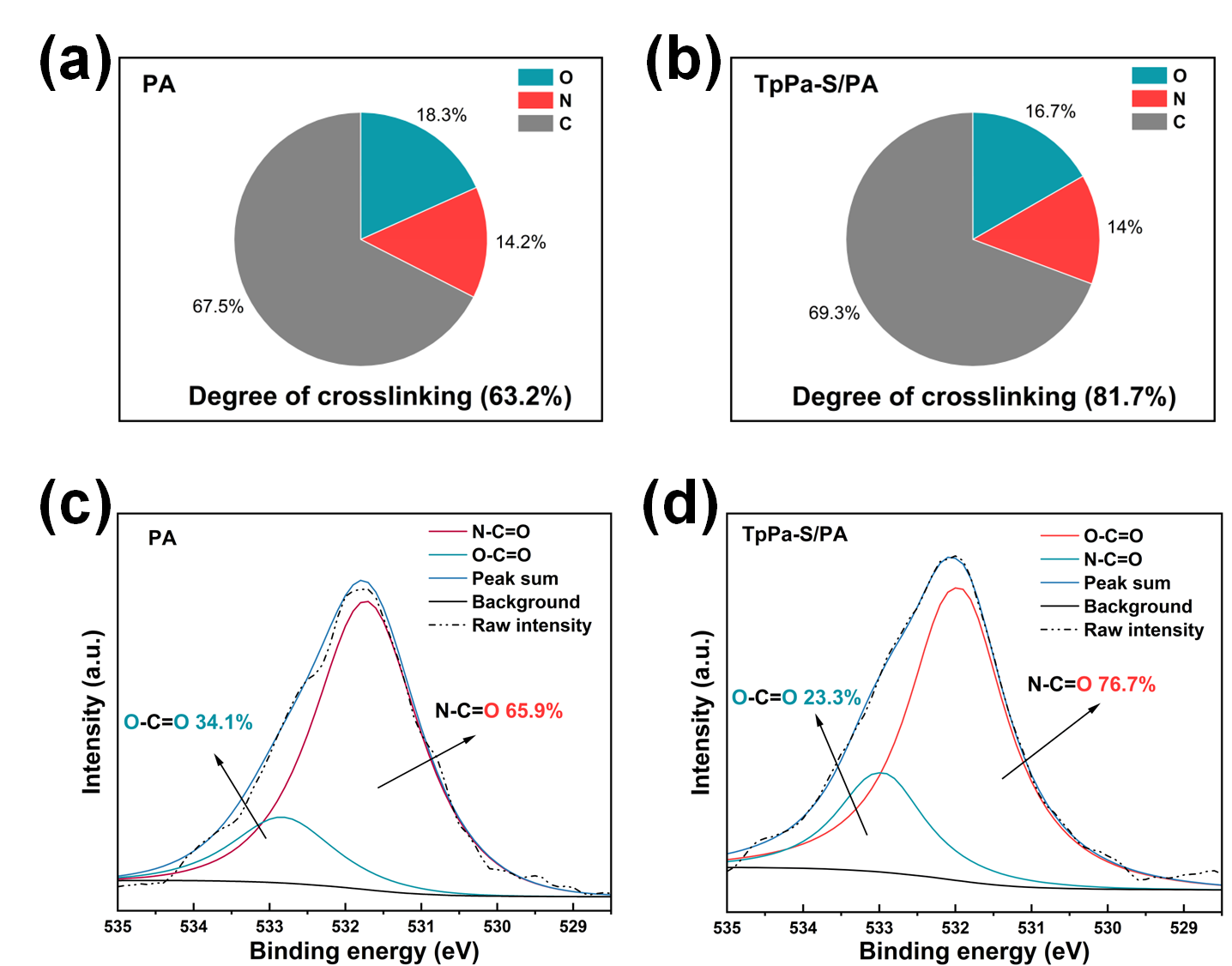


**Fig.S12** Atomic composition and cross-linking degree of (a) PA and (b) TpPa-S/PA membranes and high-resolution O1s XPS spectra of (c) PA and (d) TpPa-S/PA membranes.

**Table S3** Elemental composition, O/N ratio and degree of crosslinking of the NF membranes.


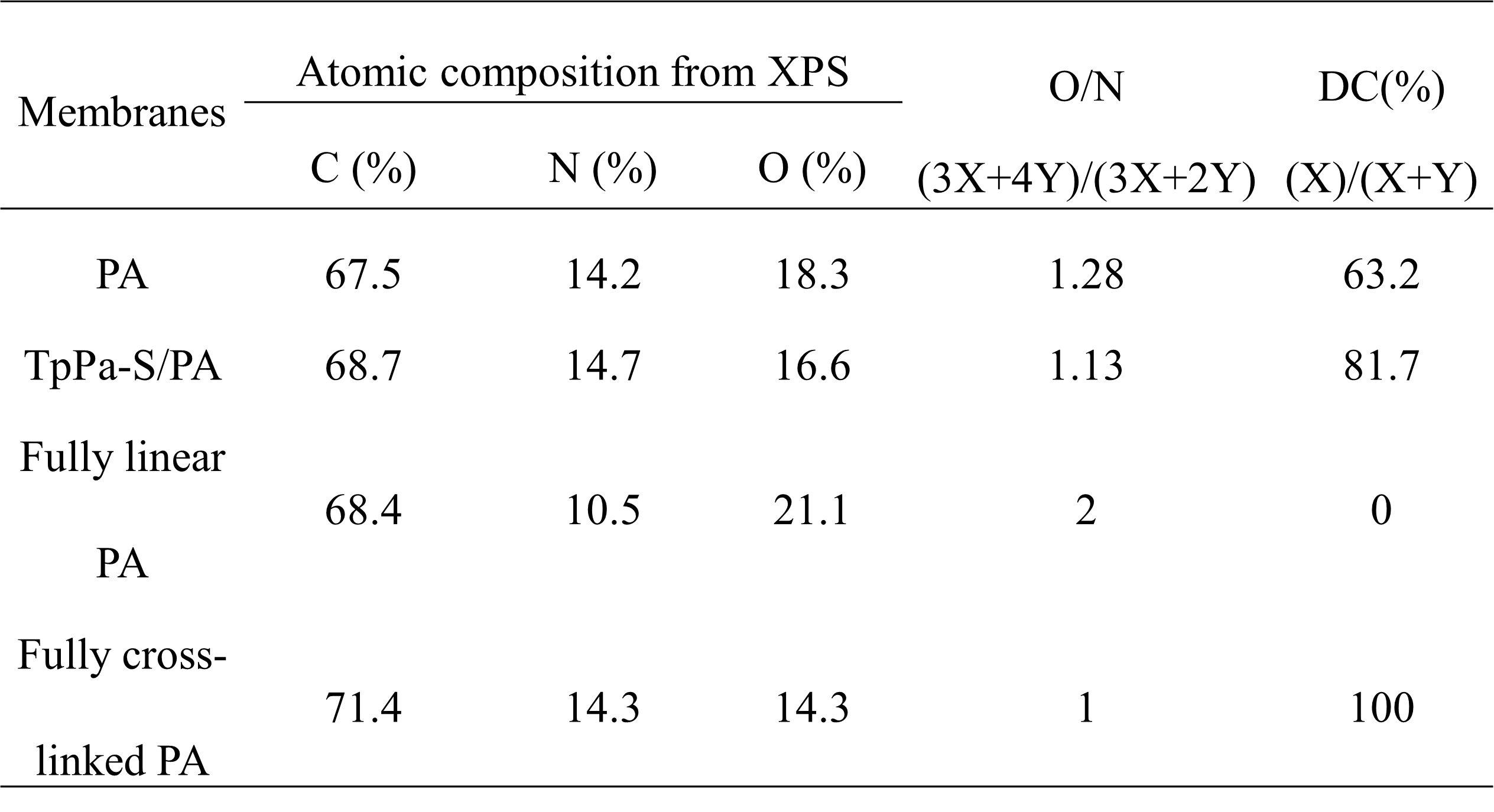


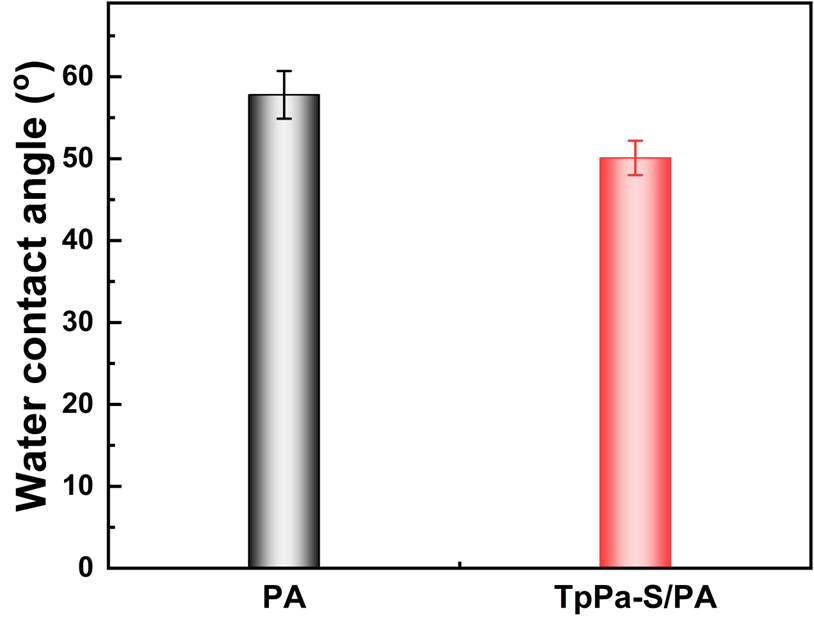


**Fig.S13** Water contact angle of the PA and TpPa-S/PA membranes.


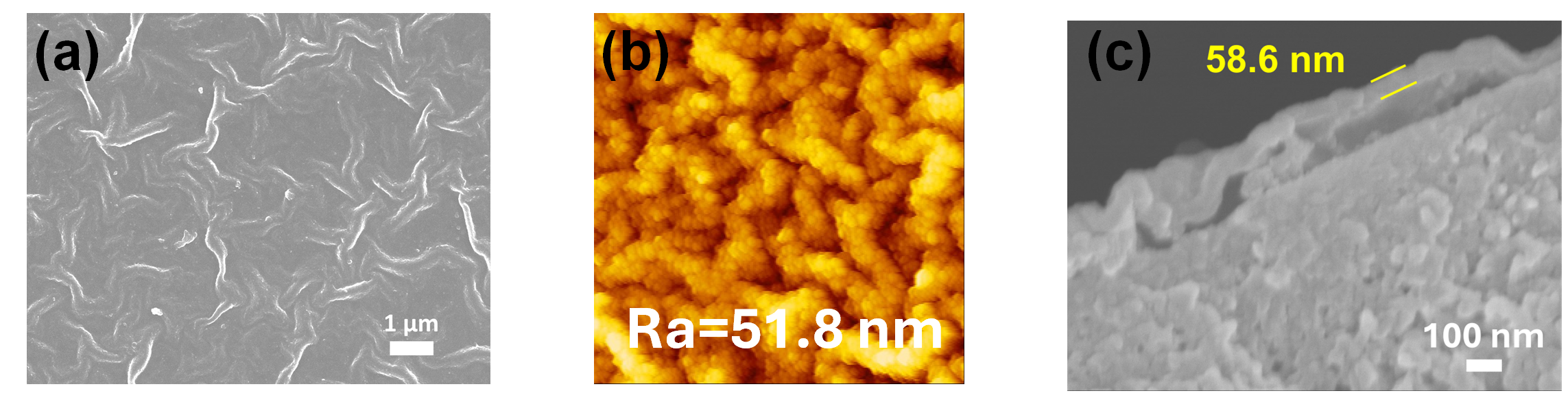


**Fig.S14** (a) SEM surface image, (b) AFM surface image and (c) SEM cross-sectional image of TpPa/PA membrane


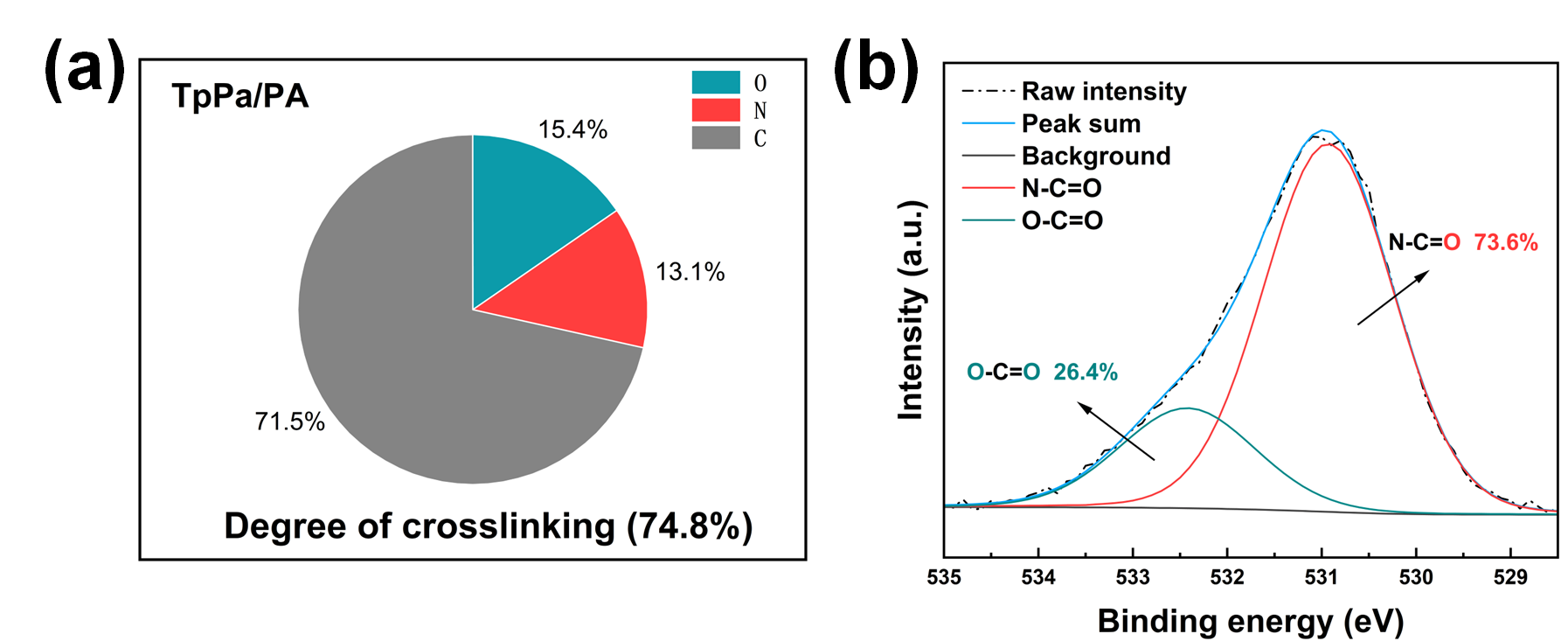


**Fig.S15** (a) Atomic composition and cross-linking degree of and (b) high-resolution O1s XPS spectra of TpPa/PA membrane.


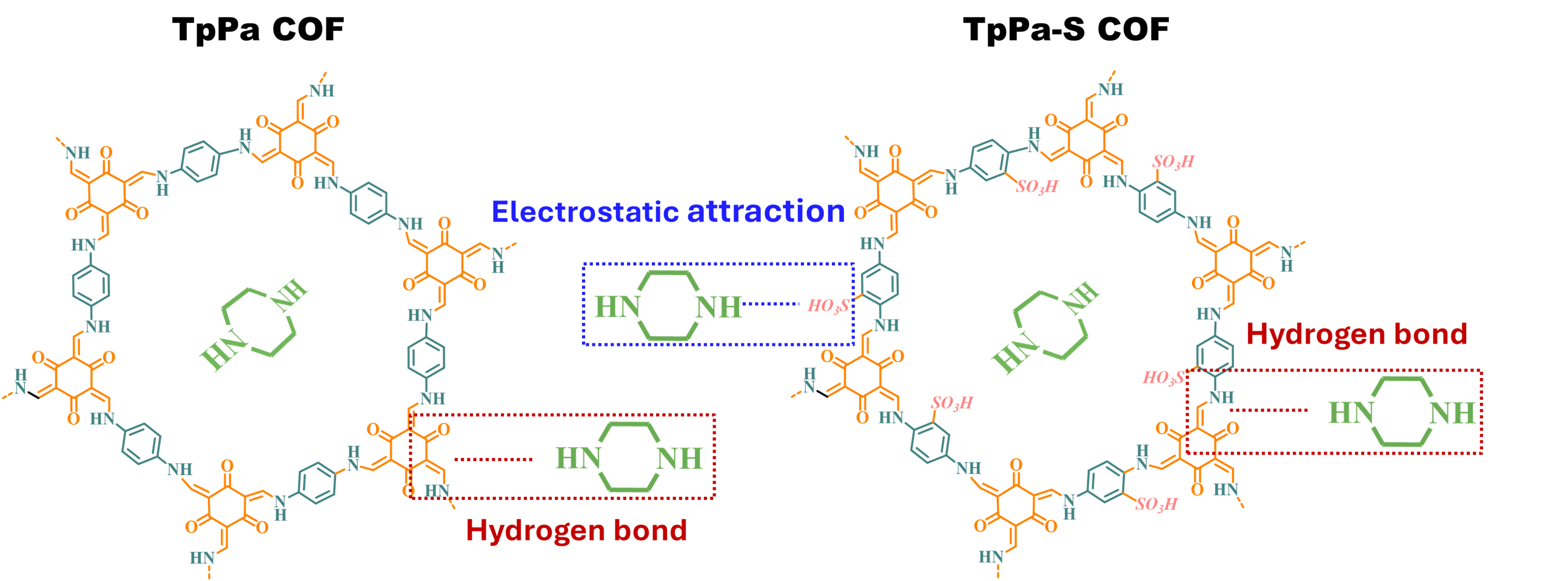


**Fig.S16** Schematic of TpPa COF and TpPa-S COF structure and nanoconfinement effect on PIP


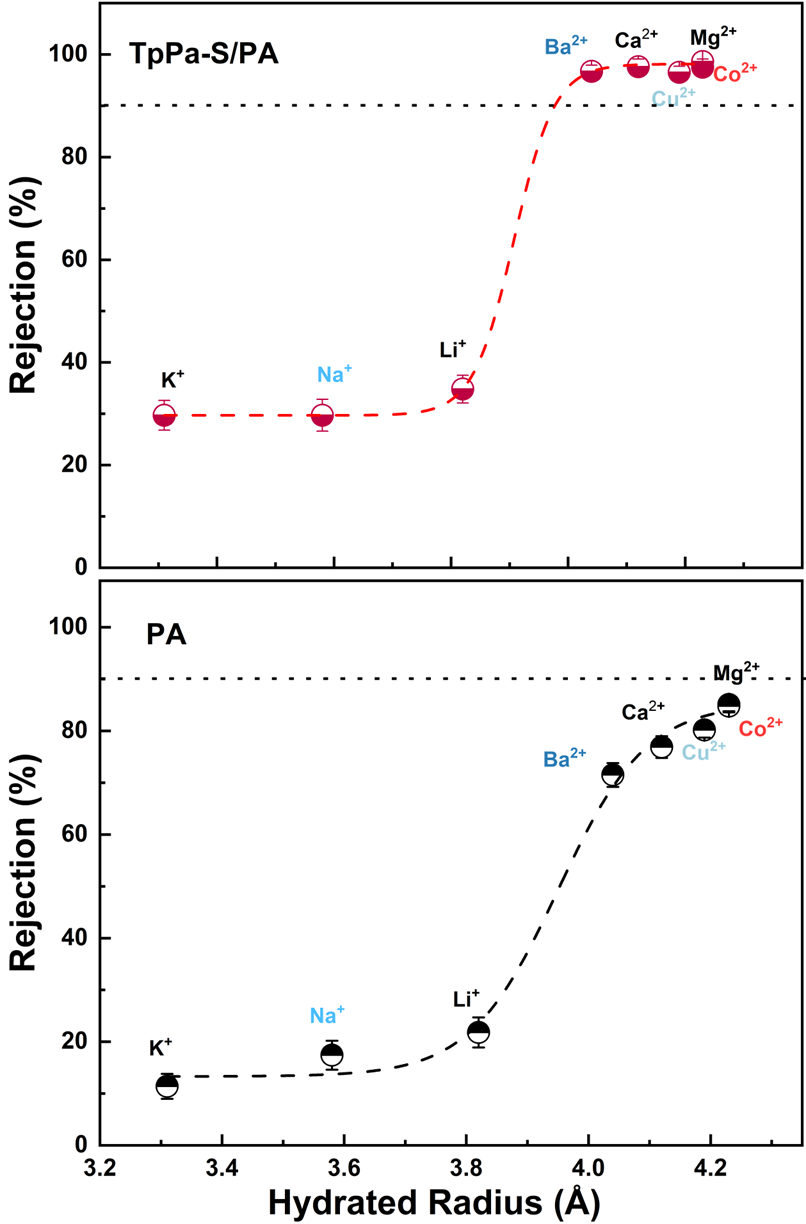


**Fig.S17** Rejection of different ions as a function of the hydrated radius for the pristine PA membranes and TpPa-S/PA membranes

**Table S4** Ionic radius, hydrated radius, hydration energy, and separation performance of ions investigated in this study. Note: data was collected from the same literature^[2]^


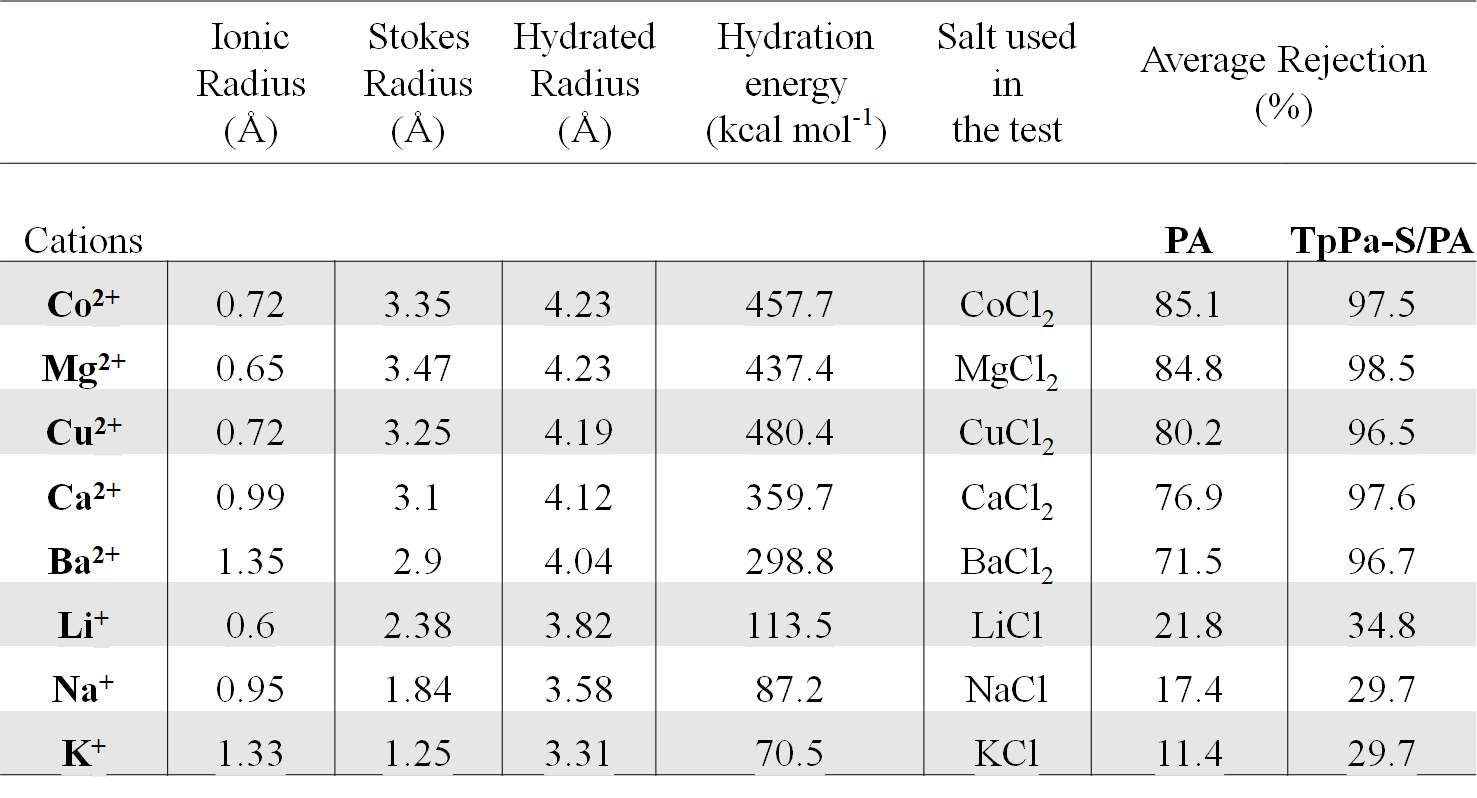


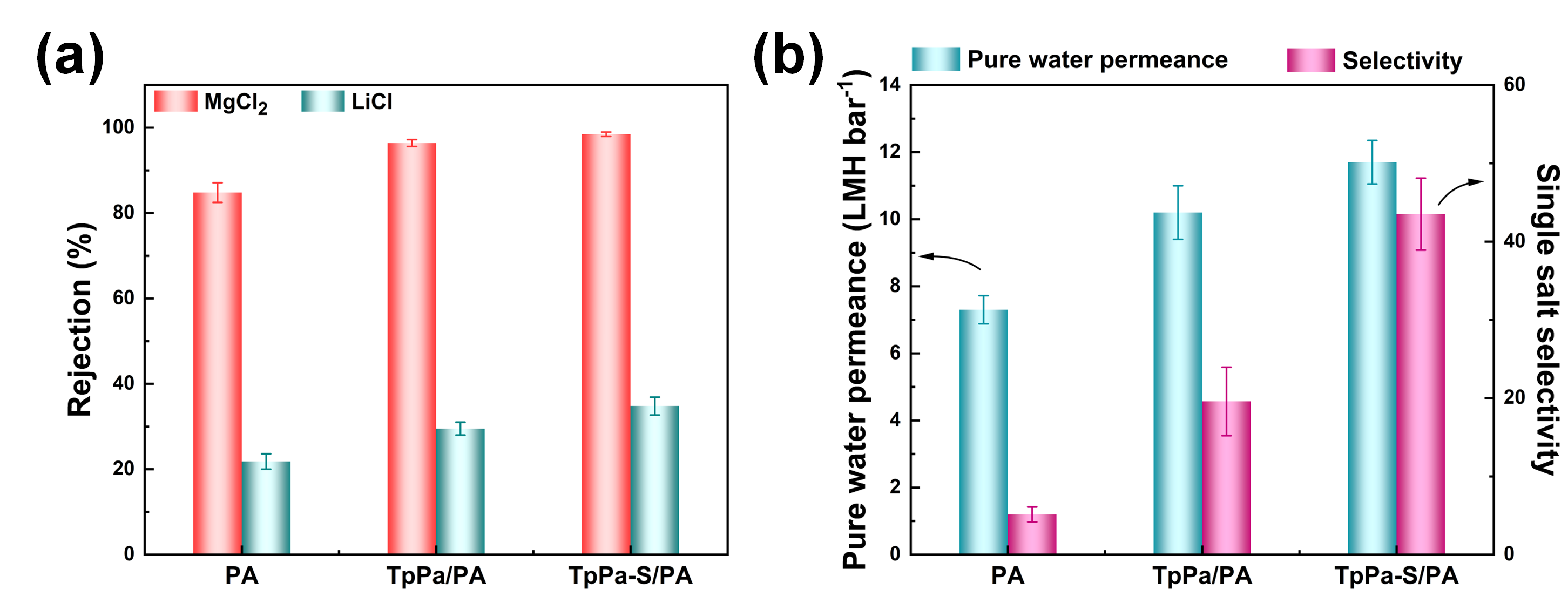


**Fig.S18** (a) Single salt rejection and (b) pure water permeance and single salt selectivity of PA membranes.

**
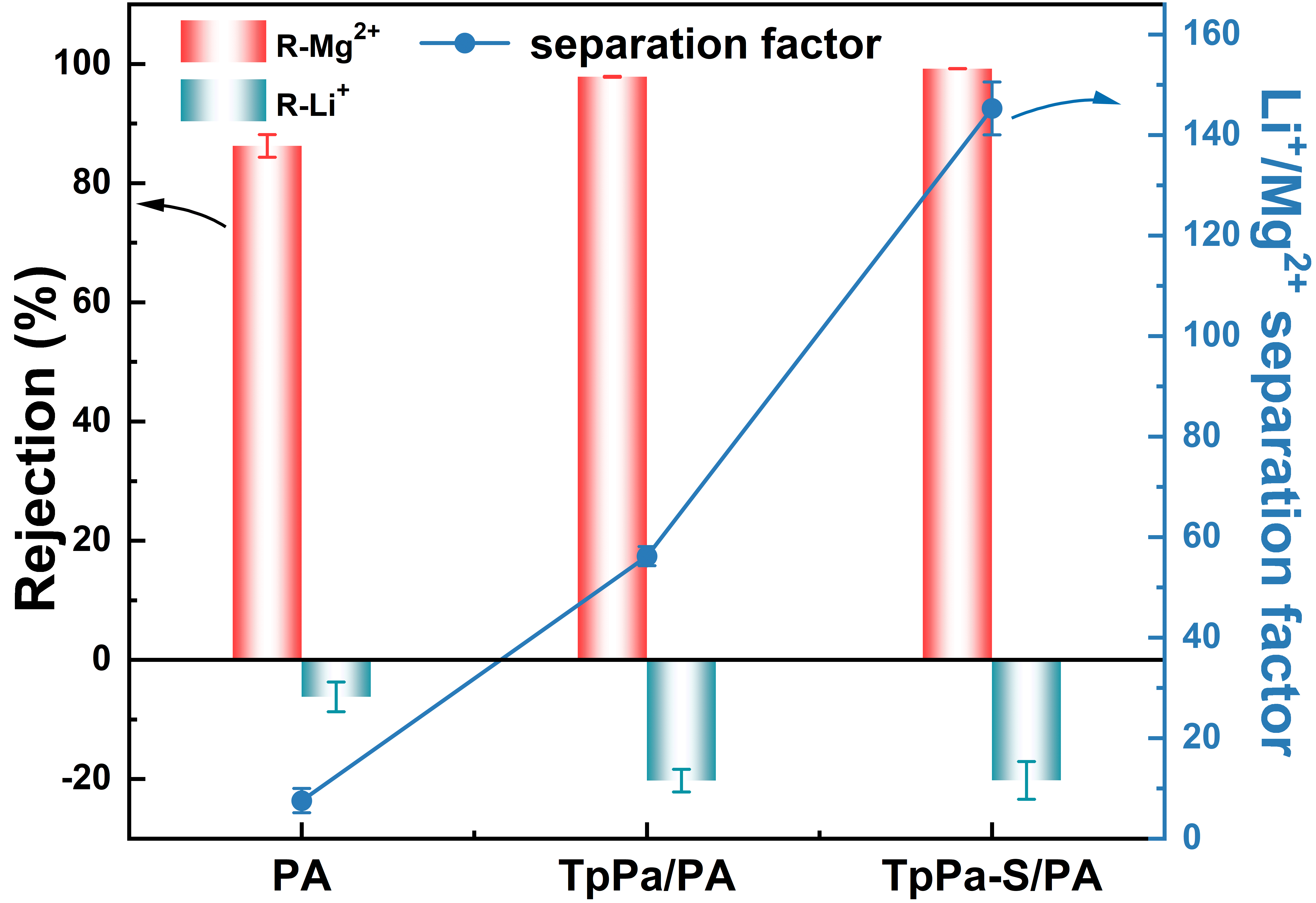
**

**Fig.S19** Mixed salt rejection and separation factor of PA membranes (Mg^2+^: Li⁺ = 20:1, 2000ppm).


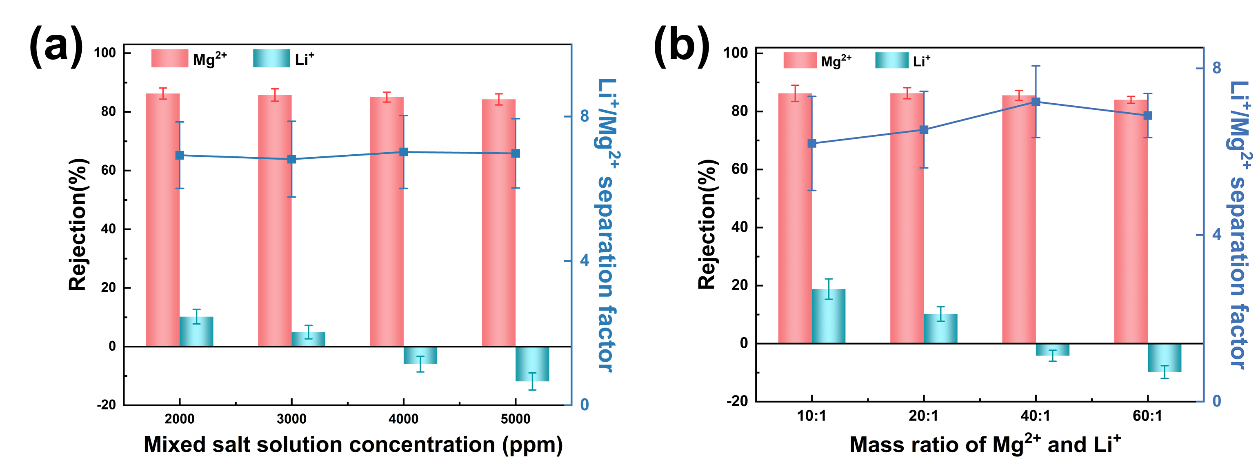


**Fig. S20** (a) Variation in separation permeance of pristine PA membrane as a function of the total concentration of mixed salts (Mg^2+^: Li⁺ = 20:1). (b) Effect of the mass ratio of Mg²⁺ to Li⁺ on separation permeance, keeping the total mixed salt concentration fixed at 2 g L^-1^.

**Table S5** The representative reported data for each type of membrane are chosen to conduct the module analysis

| Type | **Mean pore size** (Å) | **Flux**  (LMH/bar) | **P _Li_^+^**  (μm S^-1^) | **P _Mg_^+^**  (μm S^-1^) | **Separation factor** | **Reference** |
| --- | --- | --- | --- | --- | --- | --- |
| PIP-TMC/CA-PEI | 3.82 | 16 | 2.53 | 0.14 | 66.2 | ^[3]^ |
| N-CPTC–TAEA | 1.68 | 13.6 | 8.68 | 0.45 | 36.5 | ^[4]^ |
| PES/(PIP–PHF)/TMC | 3.19 | 6.7 | 1.19 | 4.76 | 13.2 | ^[5]^ |
| NF 90 | 3.60 | 3.3 | 0.11 | 1.25 | 52.6 | ^[6]^ |
| DK | 4.33 | 1.1 | 0.32 | 0.06 | 22.0 | ^[7]^ |
| DL | 4.61 | 2.1 | 0.33 | 0.53 | 3.5 | ^[8]^ |
| PIP-TMC/POP | 2.15 | 8.4 | 1.26 | 2.37 | 78.6 | ^[9]^ |
| PIP-TMC | 2.54 | 12.28 | 1.05 | 0.19 | 78.27 | ^[10]^ |
| PSF-160A-PA | 1.32 | 31.04 | 4.15 | 1.98 | 39.64 | ^[11]^ |
| NF 270 | 4.20 | 19.7 | 1.64 | 3.26 | 6.1 | ^[12]^ |
| PIP-TMC/ZIF-8 | 2.17 | 4.47 | 3.72 | 4.17 | 78.6 | ^[13]^ |
| A-PA/BA-G4D | 1.89 | 23.29 | 3.27 | 0.47 | 116.8 | ^[14]^ |
| PIP-TMC/0.02% AB2 | 3.95 | 12.73 | 1.11 | 0.87 | 72.7 | ^[15]^ |
| NF-HACC-0.3 | 3.51 | 15.7 | 2.19 | 0.21 | 115 | ^[16]^ |
| NH_2_-B15C5/PIP-TMC | 3.1 | 8.22 | 0.21 | 0.268 | 18.72 | ^[17]^ |
| SIAIP | 4.7 | 11.06 | 1.72 | 0.98 | 42.15 | ^[18]^ |
| PIP-TMC/COF | 2.60 | 11.5 | 1.80 | 0.82 | 145.3 | This work |

**
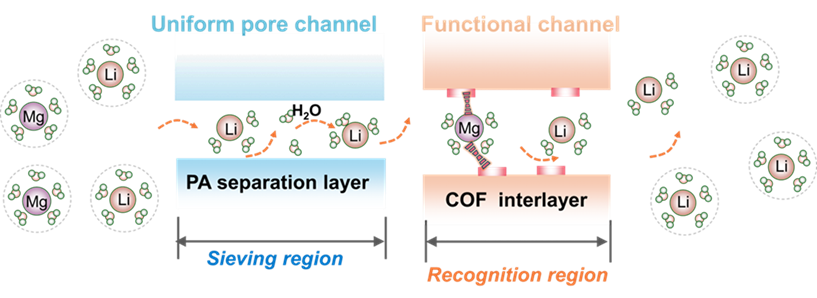
**

**Fig.S21** Possible mechanism of TpPa-S/PA membrane for precise separation of Mg^2+^ and Li^+^_._

In contrast, as shown in Fig.S22, the reverse interfacial polymerization was used to prepare another TpPa-S COF layer, except the order of the two monomer solutions was reversed, while other conditions (concentration, reaction time) were maintained constant. The resulting modified PSF membrane was designated as RTpPa-S/PSF, where "R" indicates reverse interfacial polymerization.


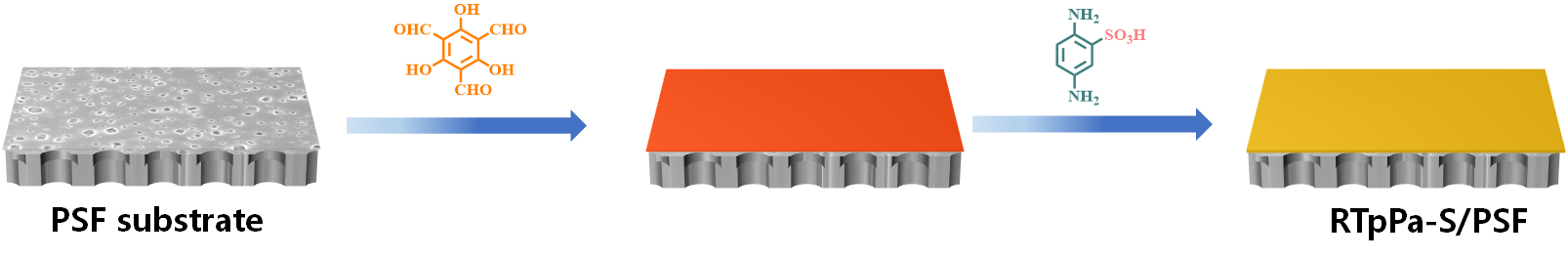


**Fig. S22** Schematic of RTpPa-S COF interlayer preparation


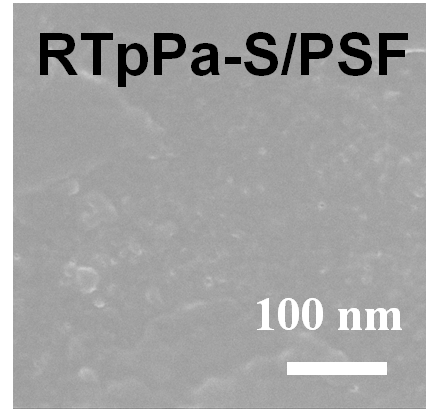


**Fig. S23** SEM surface image of RTpPa-S/PSF


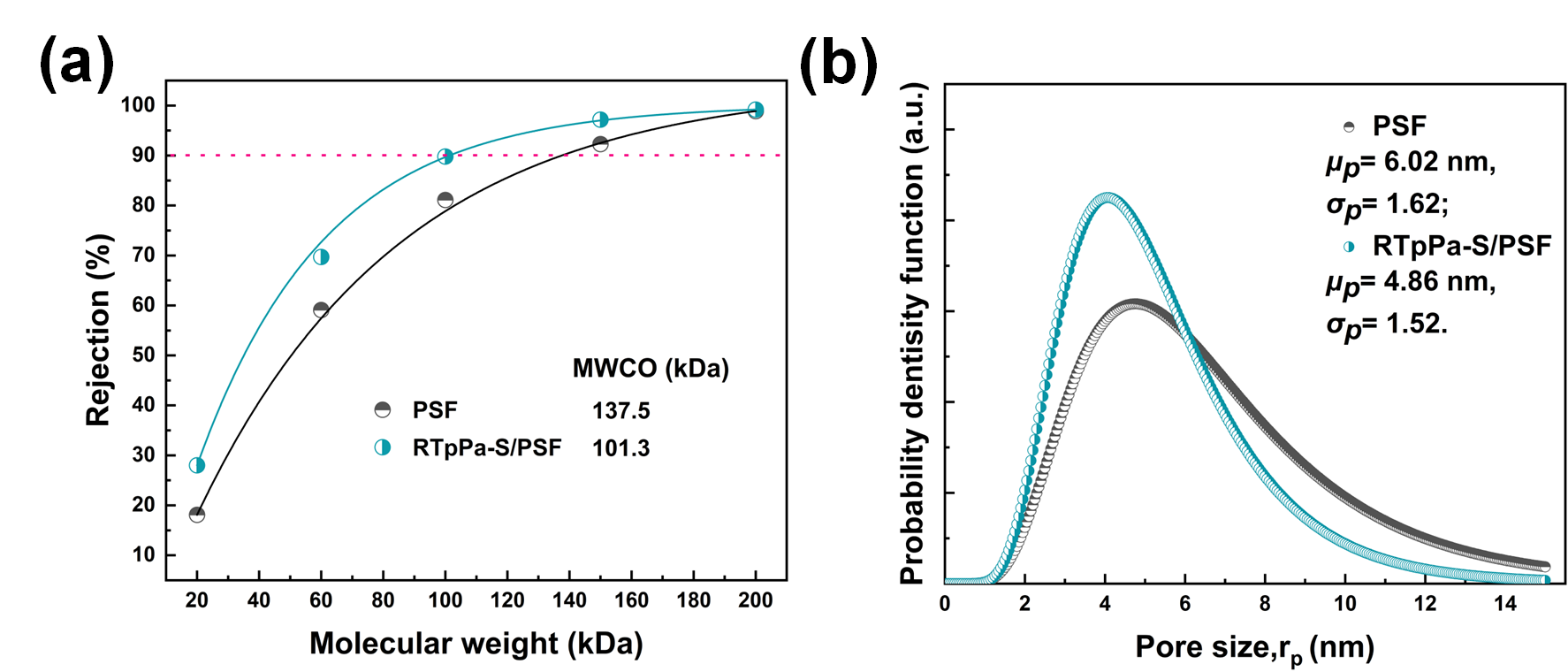


**Fig. S24** (a) MWCO and (b) pore size distribution of the PSF substrates with and without the RTpPa-S COF layers.


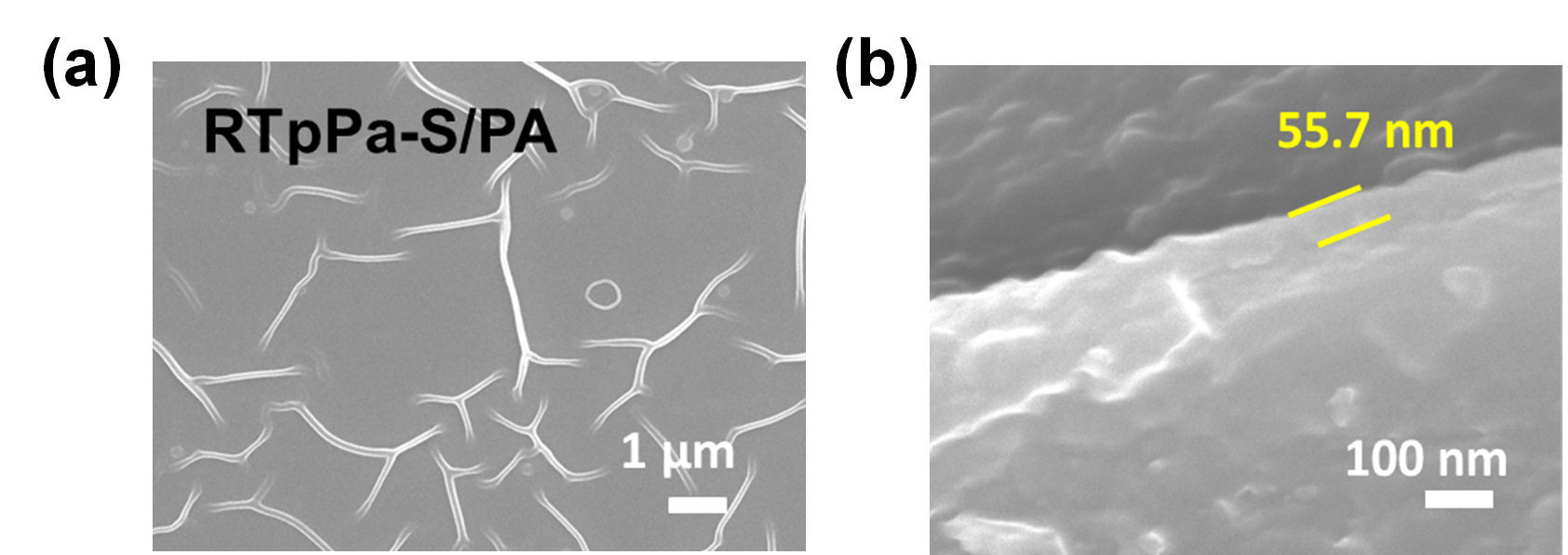


**Fig. S25** The SEM surface morphology (a) and the cross-section morphology of the RTpPa-S/PA membranes

**
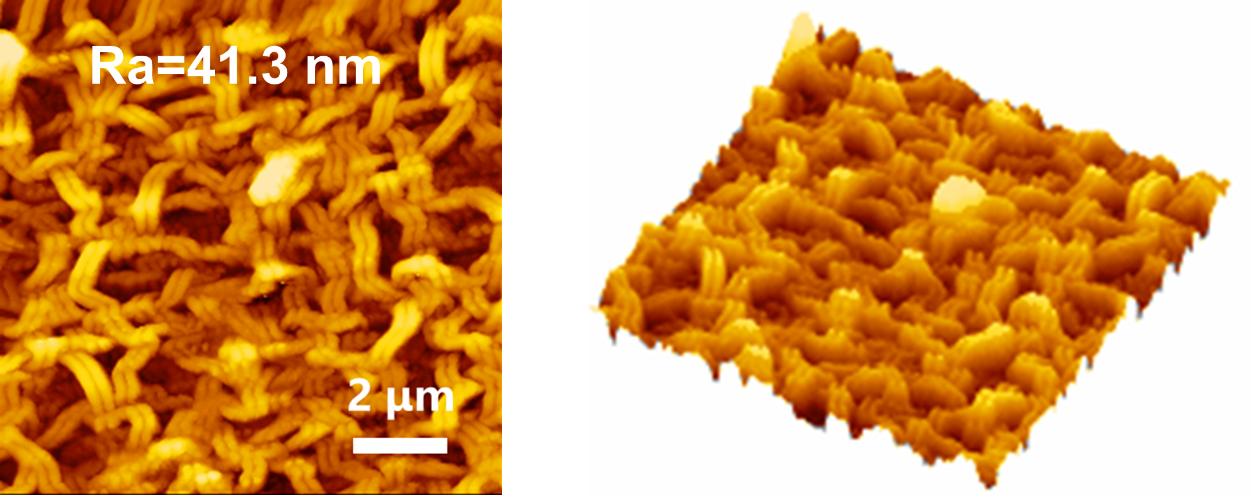
**

**Fig. S26** The AFM surface morphology of the RTpPa-S/PA membrane


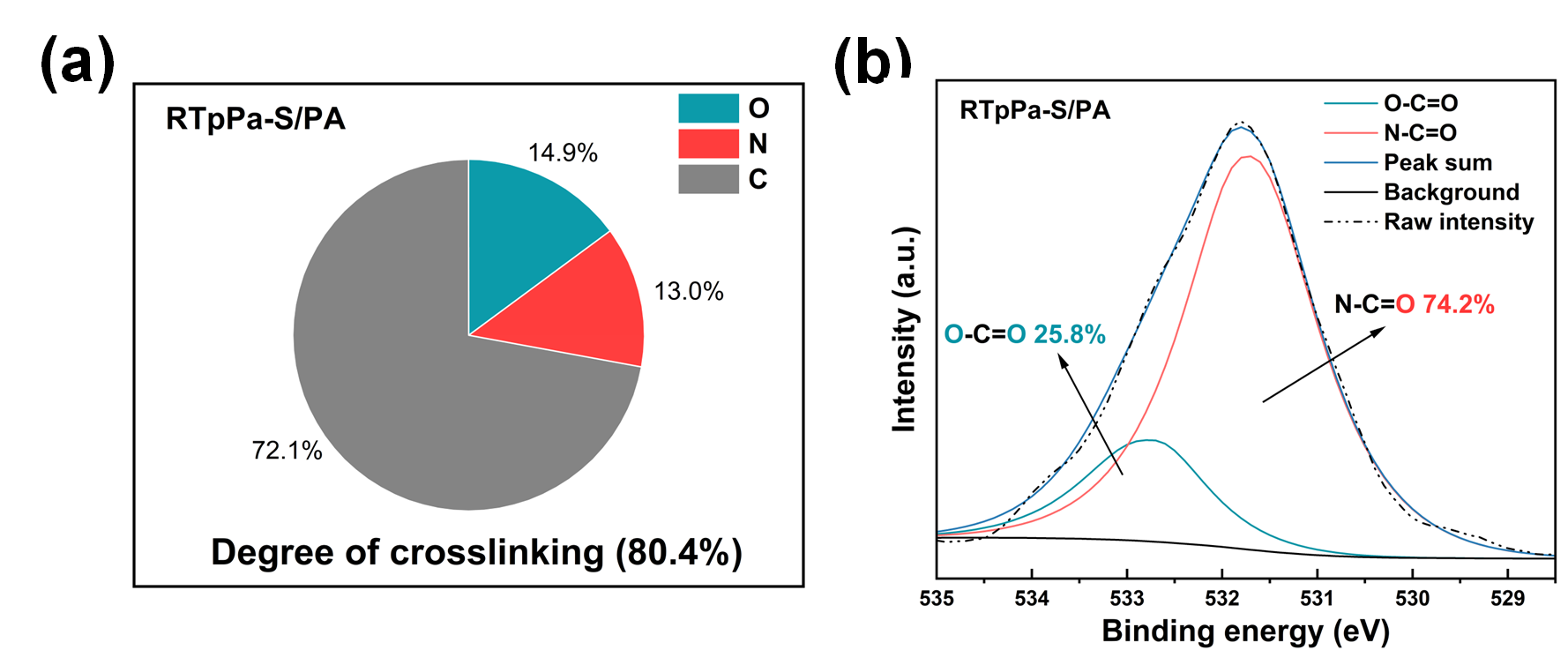


**Fig. S27** (a)Atomic composition and cross-linking degree and (b) high-resolution O1s XPS spectra of RTpPa-S/PA membrane.


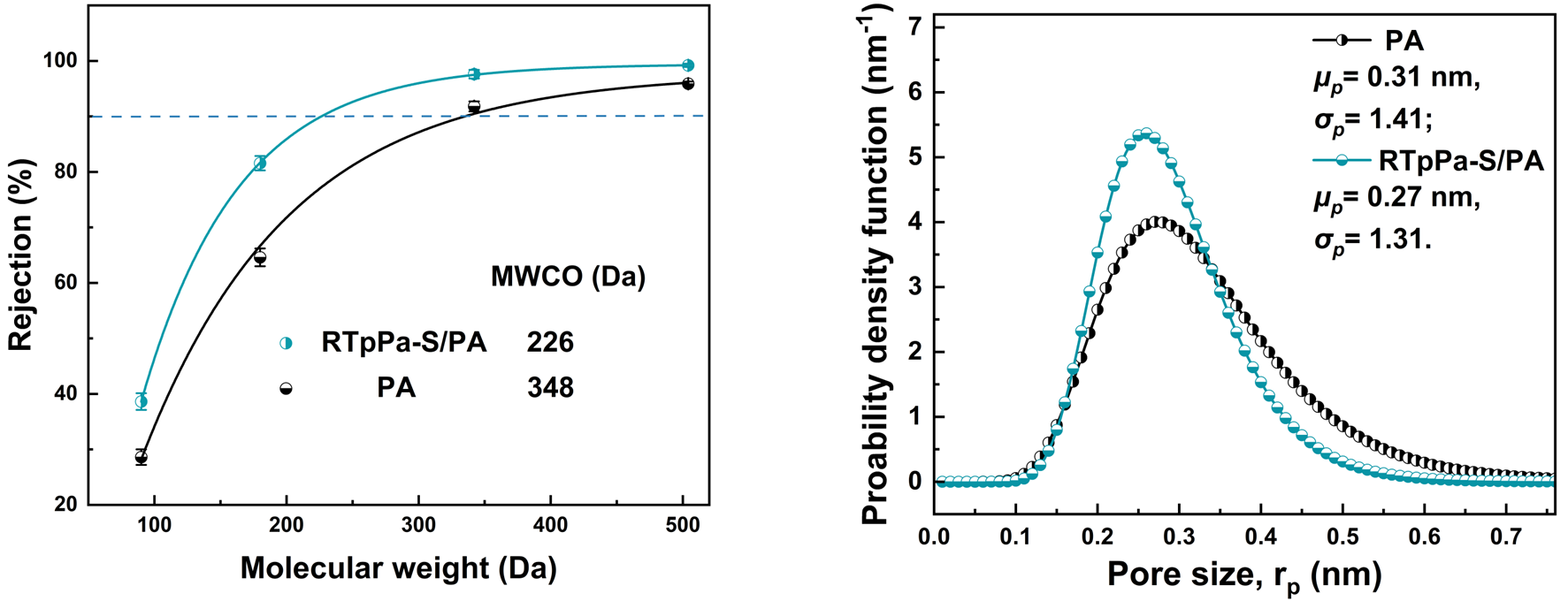


**Fig. S28** (a) MWCOs and (b) pore size density distribution function of the PA and RTpPa-S/PA membranes.

**
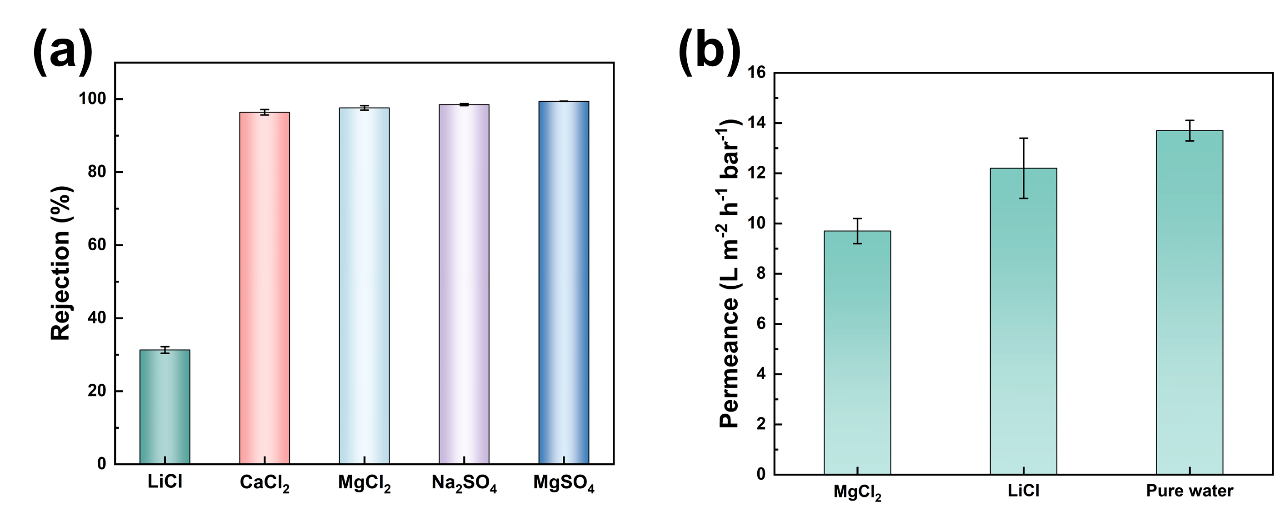
**

**Fig. S29** (a) Different salt solution rejection and (b) permeance of RTpPa-S/PA membrane


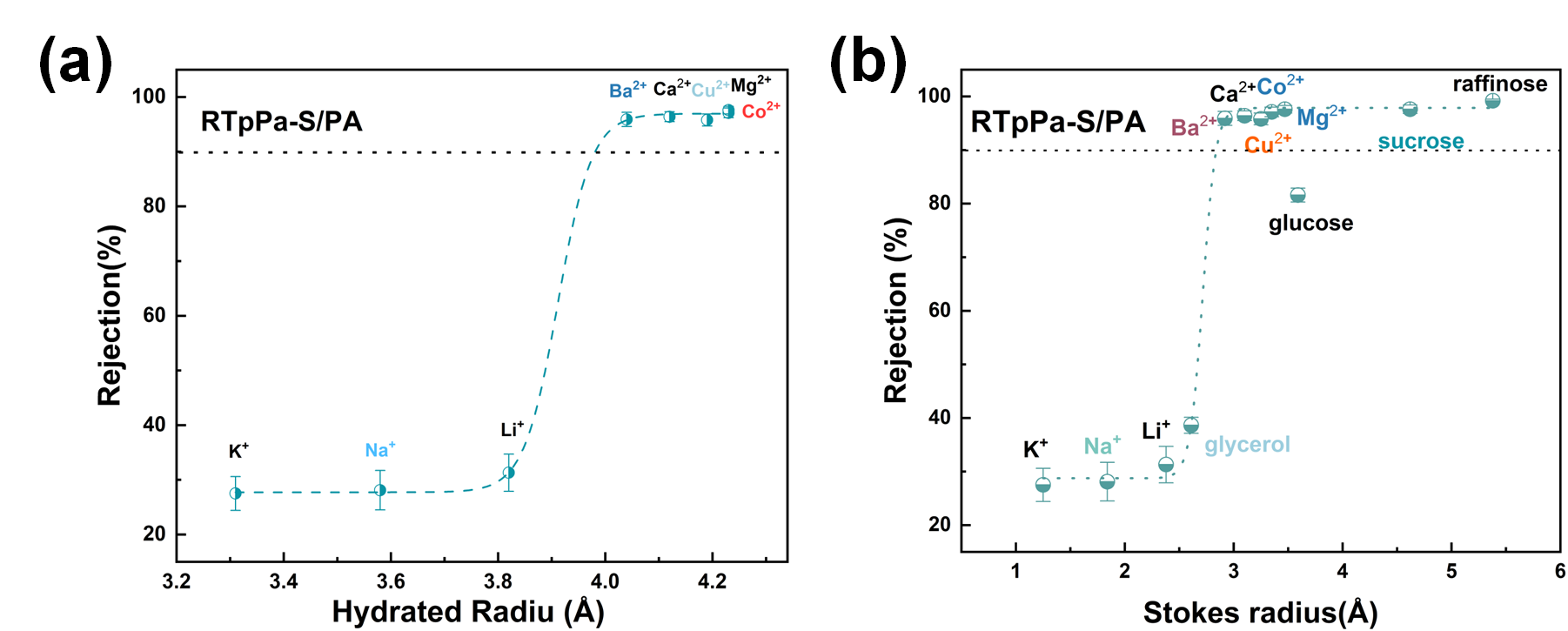


**Fig. S30** Rejection of different solutes as a function of (a) hydrated radius and (b) the Stokes radius for the RTpPa-S/PA membrane

**References**

[1] S. Yuan, G. Zhang, J. Zhu, N. Mamrol, S. Liu, Z. Mai, P. V. Puyvelde, B. V. der Bruggen, *J. Mater. Chem. A* **2020**, *8*, 3238.

[2] Y. Liang, Y. Zhu, C. Liu, K.-R. Lee, W.-S. Hung, Z. Wang, Y. Li, M. Elimelech, J. Jin, S. Lin, *Nat. Commun.* **2020**, *11*, 2015.

[3] K. Chen, S. Zhao, H. Lan, T. Xie, H. Wang, Y. Chen, P. Li, H. Sun, Q. J. Niu, C. Yang, *J. Membr. Sci.* **2022**, *660*, 120860.

[4] B. Yuan, S. Zhao, S. Xu, N. Wang, P. Hu, K. Chen, J. Jiang, J. Cui, X. Zhang, M. You, Q. J. Niu, *J. Membr. Sci.* **2022**, *660*, 120839.

[5] Q. Shen, S.-J. Xu, Z.-L. Xu, H.-Z. Zhang, Z.-Q. Dong, *J. Appl. Polym. Sci.* **2019**, *136*, 48029.

[6] B. K. Pramanik, M. B. Asif, S. Kentish, L. D. Nghiem, F. I. Hai, *J. Environ. Chem. Eng.* **2019**, *7*, 103395.

[7] Y. Li, Y. Zhao, H. Wang, M. Wang, *Desalination* **2019**, *468*, 114081.

[8] S.-Y. Sun, L.-J. Cai, X.-Y. Nie, X. Song, J.-G. Yu, *J. Water Process Eng.* **2015**, *7*, 210.

[9] S. Zhao, W. Cui, Q. Shen, Z. Yao, C. Fang, L. Zhang, L. Zhu, *J. Membr. Sci.* **2024**, *690*, 122207.

[10] G. Zhao, H. Gao, Z. Qu, H. Fan, H. Meng, *Nat. Commun.* **2023**, *14*, 7624.

[11] B. Yuan, M. Wang, M. Wu, D. Yang, K. Zhang, S. Zhao, Y. Zhang, P. Hu, M. You, S. Zhao, K. Chen, X. Zhang, J. Jiang, X. Lou, Q. J. Niu, *J. Membr. Sci.* **2024**, *701*, 122743.

[12] R. Wang, R. He, T. He, M. Elimelech, S. Lin, *Nature Water* **2023**, *1*, 291.

[13] B. Yuan, N. Wang, S. Zhao, P. Hu, J. Jiang, J. Cui, X. Zhang, M. You, X. Lou, Q. J. Niu, *Desalination* **2022**, *538*, 115929.

[14] B. Yuan, Y. Zhang, P. Qi, D. Yang, P. Hu, S. Zhao, K. Zhang, X. Zhang, M. You, J. Cui, J. Jiang, X. Lou, Q. J. Niu, *Nat. Commun.* **2024**, *15*, 471.

[15] P. Hu, B. Yuan, Q. J. Niu, K. Chen, Z. Xu, B. Tian, X. Zhang, *Desalination* **2022**, *527*, 115553.

[16] T. Zhang, Y. Chen, Q. Yu, H. Sun, K. Chen, H. Ye, S. Tang, H. Zhang, P. Li, Q. Jason Niu, *Appl. Surf. Sci.* **2023**, *616*, 156434.

[17] C. Jiang, S. Bai, J. Li, M. Wang, Y. Zhou, Y. Hou, *Journal of Membrane Science* **2024**, 123372.

[18] R. Jia, X.-G. Jin, Z.-L. Xu, L.-K. Wu, Y.-H. Tong, H.-X. Li, H.-H. Ping, X.-H. Ma, S.-J. Xu, *J. Membr. Sci.* **2024**, *712*, 123235.
